# Supplementary material for: Segregation analysis of 17,425 population-based breast cancer families: Evidence for genetic susceptibility and risk prediction
Source: Am J Hum Genet. 2022 Oct 6;109(10):1777–88. doi: 10.1016/j.ajhg.2022.09.006 (PMC9606477; doi:10.1016/j.ajhg.2022.09.006)
Supplement: Document S1. Figure S1, Tables S1–S10, and supplemental acknowledgments [file mmc1.pdf]

The American Journal of Human Genetics, Volume *109*

## Supplemental information

# Segregation analysis of 17,425 population-based breast cancer families: Evidence for genetic susceptibility and risk prediction

**Shuai Li, Robert J. MacInnis, Andrew Lee, Tu Nguyen-Dumont, Leila Dorling, Sara Carvalho, Gillian S. Dite, Mitul Shah, Craig Luccarini, Qin Wang, Roger L. Milne, Mark A. Jenkins, Graham G. Giles, Alison M. Dunning, Paul D.P. Pharoah, Melissa C. Southey, Douglas F. Easton, John L. Hopper, and Antonis C. Antoniou**

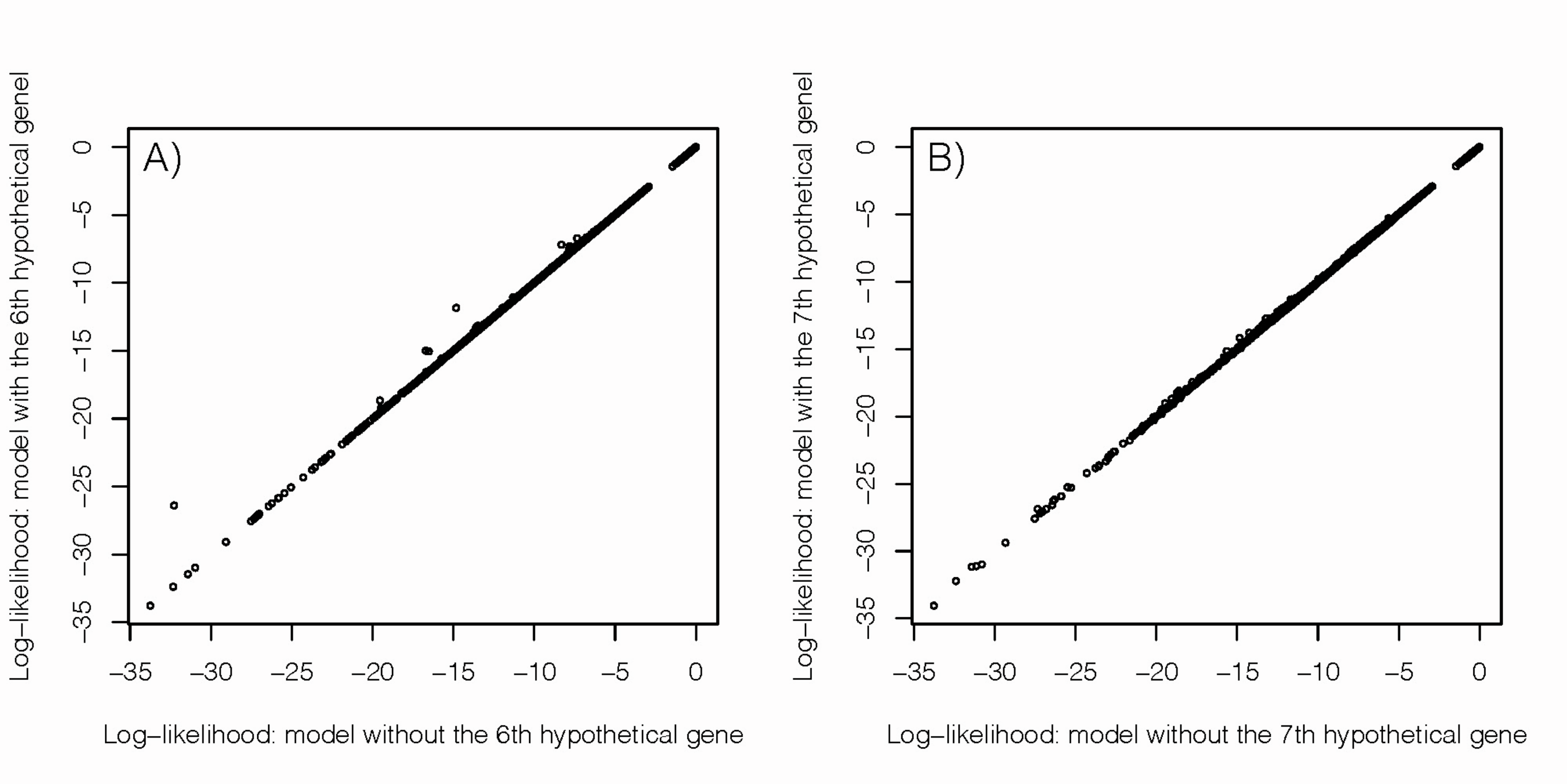


**Figure S1 Log-likelihood changes for models in favour of the sixth and seventh hypothetical gene**

A) Log-likelihood changes from the analysis of the sixth hypothetical gene. X axis is the log-likelihood of the model including *BRCA1*, *BRCA2*, *PALB2*, *CHEK2*, *ATM* and an age-constant $\sigma_P^2(t)$, and Y axis is the log-likelihood of the best fitting dominant inheritance model of the sixth hypothetical gene.

B) Log-likelihood changes from the analysis of the seventh hypothetical gene. X axis is the log-likelihood of the model including *BRCA1*, *BRCA2*, *PALB2*, *CHEK2*, *ATM*, *TP53* and an age-constant $\sigma_P^2(t)$, and Y axis is the log-likelihood of the best fitting recessive inheritance model of the seventh hypothetical gene.

**Table S1 Assumed sensitivity of the pathogenic variant test methods in the ABCFR**

| Method | Assumed sensitivity |
|---|---|
| Heteroduplex Analysis | 70% |
| Protein Truncation Test (PTT) | 90% |
| Predictive Sequencing | 100% |
| 2-Dimensional Gel Electrophoresis | 70% |
| Automated DNA Sequencing | 100% |
| Multiplex ligation-dependent probe amplification (MLPA) | 100% |
| MYRIAD DNA Sequencing | 100% |
| Manual DNA Sequencing | 100% |
| Denaturing High Performance Liquid (DHPLC) | 90% |
| PCR and Agarose Gel Electrophoresis | 70% |
| 5’Nuclease Assay (TaqMan) | 95% |
| Gene-panel test | 100% |

The test sensitivity used in analysis was the sum of the sensitivities of the test methods used, weighted by the proportional lengths of the exons screened, multiplying by 90% if MLPA had not been conducted. If MLPA had been conducted, 10% multiplying the sensitivity of MLPA weighted by the proportional lengths of the exons screened was added.

**Table S2 Age-specific breast and ovarian cancer RRs for BRCA1, BRCA2, PALB2 and CHEK2 PVs**

| Age | Breast cancer RRs | | | | Ovarian cancer RRs | |
|---|---|---|---|---|---|---|
| | *BRCA1* | *BRCA2* | *PALB2* | *CHEK2* | *BRCA1* | *BRCA2* |
| 0 | 1.0 | 1.0 | 1.0 | 1.0 | 1.0 | 1.0 |
| 1 | 1.0 | 1.0 | 1.0 | 1.0 | 1.0 | 1.0 |
| 2 | 1.0 | 1.0 | 1.0 | 1.0 | 1.0 | 1.0 |
| 3 | 1.0 | 1.0 | 1.0 | 1.0 | 1.0 | 1.0 |
| 4 | 1.0 | 1.0 | 1.0 | 1.0 | 1.0 | 1.0 |
| 5 | 1.0 | 1.0 | 1.0 | 1.0 | 1.0 | 1.0 |
| 6 | 1.0 | 1.0 | 1.0 | 1.0 | 1.0 | 1.0 |
| 7 | 1.0 | 1.0 | 1.0 | 1.0 | 1.0 | 1.0 |
| 8 | 1.0 | 1.0 | 1.0 | 1.0 | 1.0 | 1.0 |
| 9 | 1.0 | 1.0 | 1.0 | 1.0 | 1.0 | 1.0 |
| 10 | 1.0 | 1.0 | 1.0 | 1.0 | 1.0 | 1.0 |
| 11 | 1.0 | 1.0 | 1.0 | 1.0 | 1.0 | 1.0 |
| 12 | 1.0 | 1.0 | 1.0 | 1.0 | 1.0 | 1.0 |
| 13 | 1.0 | 1.0 | 1.0 | 1.0 | 1.0 | 1.0 |
| 14 | 1.0 | 1.0 | 1.0 | 1.0 | 1.0 | 1.0 |
| 15 | 1.0 | 1.0 | 1.0 | 1.0 | 1.0 | 1.0 |
| 16 | 1.0 | 1.0 | 1.0 | 1.0 | 1.0 | 1.0 |
| 17 | 1.0 | 1.0 | 1.0 | 1.0 | 1.0 | 1.0 |
| 18 | 1.0 | 1.0 | 1.0 | 1.0 | 1.0 | 1.0 |
| 19 | 1.0 | 1.0 | 1.0 | 1.0 | 1.0 | 1.0 |
| 20 | 33.0 | 20.9 | 14.6 | 3.7 | 1.0 | 1.0 |
| 21 | 33.8 | 20.7 | 14.3 | 3.6 | 1.0 | 1.0 |
| 22 | 34.6 | 20.5 | 14.0 | 3.6 | 1.0 | 1.0 |
| 23 | 35.5 | 20.3 | 13.7 | 3.5 | 1.0 | 1.0 |
| 24 | 36.4 | 20.2 | 13.4 | 3.5 | 1.0 | 1.0 |
| 25 | 37.2 | 20.0 | 13.1 | 3.4 | 1.0 | 1.0 |
| 26 | 38.2 | 19.8 | 12.9 | 3.4 | 1.0 | 1.0 |
| 27 | 39.1 | 19.6 | 12.6 | 3.3 | 1.0 | 1.0 |
| 28 | 40.0 | 19.5 | 12.3 | 3.3 | 1.0 | 1.0 |
| 29 | 41.0 | 19.3 | 12.1 | 3.2 | 1.0 | 1.0 |
| 30 | 42.0 | 19.1 | 11.8 | 3.2 | 11.1 | 1.0 |
| 31 | 38.9 | 18.3 | 11.6 | 3.1 | 13.6 | 1.0 |
| 32 | 36.0 | 17.5 | 11.3 | 3.1 | 16.5 | 1.0 |
| 33 | 33.3 | 16.7 | 11.1 | 3.1 | 20.2 | 1.0 |
| 34 | 30.8 | 16.0 | 10.9 | 3.0 | 24.6 | 1.0 |
| 35 | 28.5 | 15.3 | 10.6 | 3.0 | 30.0 | 1.0 |
| 36 | 26.4 | 14.6 | 10.4 | 2.9 | 36.6 | 1.0 |
| 37 | 24.4 | 14.0 | 10.2 | 2.9 | 44.6 | 1.0 |
| 38 | 22.6 | 13.4 | 10.0 | 2.8 | 54.4 | 1.0 |
| 39 | 20.9 | 12.8 | 9.8 | 2.8 | 66.4 | 1.0 |
| 40 | 19.3 | 12.2 | 9.6 | 2.8 | 81.0 | 1.0 |
| 41 | 18.7 | 11.8 | 9.4 | 2.7 | 75.5 | 1.3 |
| 42 | 18.1 | 11.4 | 9.2 | 2.7 | 70.4 | 1.6 |
| 43 | 17.5 | 11.0 | 9.0 | 2.6 | 65.7 | 2.1 |
| 44 | 17.0 | 10.6 | 8.8 | 2.6 | 61.3 | 2.6 |
| 45 | 16.4 | 10.2 | 8.6 | 2.6 | 57.2 | 3.4 |
| 46 | 15.9 | 9.8 | 8.4 | 2.5 | 53.3 | 4.3 |
| 47 | 15.4 | 9.5 | 8.3 | 2.5 | 49.7 | 5.5 |
| 48 | 14.9 | 9.1 | 8.1 | 2.4 | 46.4 | 7.0 |
| 49 | 14.4 | 8.8 | 7.9 | 2.4 | 43.3 | 8.9 |
| 50 | 14.0 | 8.5 | 7.8 | 2.4 | 40.4 | 11.3 |
| 51 | 13.6 | 8.5 | 7.6 | 2.3 | 39.7 | 14.4 |

| | | | | | | |
|---|---|---|---|---|---|---|
| 52 | 13.1 | 8.6 | 7.4 | 2.3 | 39.1 | 18.4 |
| 53 | 12.7 | 8.7 | 7.3 | 2.3 | 38.4 | 23.5 |
| 54 | 12.3 | 8.7 | 7.1 | 2.2 | 37.8 | 29.9 |
| 55 | 12.0 | 8.8 | 7.0 | 2.2 | 37.2 | 28.2 |
| 56 | 11.6 | 8.8 | 6.8 | 2.2 | 36.6 | 26.6 |
| 57 | 11.2 | 8.9 | 6.7 | 2.1 | 36.0 | 25.2 |
| 58 | 10.9 | 8.9 | 6.6 | 2.1 | 35.4 | 23.8 |
| 59 | 10.5 | 9.0 | 6.4 | 2.1 | 34.8 | 18.6 |
| 60 | 10.2 | 9.1 | 6.3 | 2.0 | 34.2 | 14.5 |
| 61 | 9.9 | 9.4 | 6.2 | 2.0 | 33.7 | 11.4 |
| 62 | 9.6 | 9.8 | 6.0 | 2.0 | 33.1 | 8.9 |
| 63 | 9.3 | 10.2 | 5.9 | 2.0 | 32.6 | 7.0 |
| 64 | 9.0 | 10.7 | 5.8 | 1.9 | 32.0 | 5.4 |
| 65 | 8.7 | 11.1 | 5.7 | 1.9 | 31.5 | 4.3 |
| 66 | 8.5 | 11.6 | 5.6 | 1.9 | 31.0 | 3.3 |
| 67 | 8.2 | 12.1 | 5.4 | 1.8 | 30.5 | 2.6 |
| 68 | 7.9 | 12.6 | 5.3 | 1.8 | 30.0 | 2.0 |
| 69 | 7.7 | 13.1 | 5.2 | 1.8 | 29.5 | 1.6 |
| 70 | 7.5 | 13.1 | 5.1 | 1.8 | 29.0 | 1.6 |
| 71 | 7.2 | 13.1 | 5.0 | 1.7 | 28.5 | 1.6 |
| 72 | 7.0 | 13.1 | 4.9 | 1.7 | 28.1 | 1.6 |
| 73 | 6.8 | 13.1 | 4.8 | 1.7 | 27.6 | 1.6 |
| 74 | 6.6 | 13.1 | 4.7 | 1.7 | 27.2 | 1.6 |
| 75 | 6.4 | 13.1 | 4.6 | 1.6 | 26.7 | 1.6 |
| 76 | 6.2 | 13.1 | 4.5 | 1.6 | 26.3 | 1.6 |
| 77 | 6.0 | 13.1 | 4.4 | 1.6 | 25.9 | 1.6 |
| 78 | 5.8 | 13.1 | 4.3 | 1.6 | 25.4 | 1.6 |
| 79 | 5.6 | 13.1 | 4.2 | 1.5 | 25.0 | 1.6 |

**Table S3 Number of ABCFR relatives found to be have PVs by the diagnosis age and PV status of the proband a**

| Gene | Number of relatives and families | | Age groups of the probands (years) | | | | | | | | | | | |
|---|---|---|---|---|---|---|---|---|---|---|---|---|---|---|
| | | | Case families | | | | | | Control families | | | | | |
| | | | <30 | 30-39 | 40-49 | 50-59 | 60-69 | Total | <30 | 30-39 | 40-49 | 50-59 | 60-69 | Total |
| *BRCA1* | Number of relatives | | 1 | 27 | 22 | 3 | 0 | 53 | 0 | 1 | 2 | 0 | 0 | 3 |
| | Number of families where the relatives come from | Probands with PVs | 1 | 17 | 9 | 1 | 0 | 28 | 0 | 1 | 1 | 0 | 0 | 2 |
| | | Probands without PVs | 0 | 0 | 1 | 1 | 0 | 2 | 0 | 0 | 0 | 0 | 0 | 0 |
| | | Probands untested | 0 | 0 | 0 | 0 | 0 | 0 | 0 | 0 | 0 | 0 | 0 | 0 |
| *BRCA2* | Number of relatives | | 4 | 25 | 7 | 5 | 0 | 41 | 0 | 0 | 0 | 0 | 0 | 0 |
| | Number of families where the relatives come from | Probands with PVs | 3 | 14 | 2 | 0 | 0 | 19 | 0 | 0 | 0 | 0 | 0 | 0 |
| | | Probands without PVs | 0 | 0 | 3 | 2 | 0 | 5 | 0 | 0 | 0 | 0 | 0 | 0 |
| | | Probands untested | 0 | 2 | 0 | 0 | 0 | 2 | 0 | 0 | 0 | 0 | 0 | 0 |
| *PALB2* | Number of relatives | | 3 | 2 | 7 | 1 | 0 | 13 | 0 | 0 | 0 | 0 | 0 | 0 |
| | Number of families where the relatives come from | Probands with PVs | 1 | 1 | 3 | 1 | 0 | 6 | 0 | 0 | 0 | 0 | 0 | 0 |
| | | Probands without PVs | 0 | 0 | 2 | 0 | 0 | 2 | 0 | 0 | 0 | 0 | 0 | 0 |
| | | Probands untested | 0 | 0 | 0 | 0 | 0 | 0 | 0 | 0 | 0 | 0 | 0 | 0 |
| *CHEK2* | Number of relatives | | 4 | 6 | 6 | 2 | 0 | 18 | 0 | 0 | 0 | 0 | 0 | 0 |
| | Number of families where the relatives come from | Probands with PVs | 2 | 4 | 4 | 0 | 0 | 10 | 0 | 0 | 0 | 0 | 0 | 0 |
| | | Probands without PVs | 0 | 1 | 0 | 2 | 0 | 3 | 0 | 0 | 0 | 0 | 0 | 0 |
| | | Probands untested | 0 | 0 | 0 | 0 | 0 | 0 | 0 | 0 | 0 | 0 | 0 | 0 |
| *ATM* | Number of relatives | | 0 | 3 | 3 | 7 | 1 | 14 | 0 | 1 | 0 | 1 | 0 | 2 |
| | Number of families where the relatives come from | Probands with PVs | 0 | 0 | 1 | 2 | 1 | 4 | 0 | 0 | 0 | 1 | 0 | 1 |
| | | Probands without PVs | 0 | 3 | 2 | 2 | 0 | 7 | 0 | 1 | 0 | 0 | 0 | 1 |
| | | Probands untested | 0 | 0 | 0 | 0 | 0 | 0 | 0 | 0 | 0 | 0 | 0 | 0 |

[a] 2 relatives had a PV in two genes: case families – one relative (proband aged 30-39 years) in *BRCA2* and *CHEK2*; control families – one relative (proband aged 30-39 years) in *BRCA1* and *ATM*

**Table S4 Number of relatives diagnosed with breast or ovarian cancer by study and the diagnosis age and PV status of the proband**

| PV status | Relative type | Cancer site | ABCFR case families | | | | | | ABCFR control families | | | | | | SEARCH | | | | | | |
|---|---|---|---|---|---|---|---|---|---|---|---|---|---|---|---|---|---|---|---|---|---|
| | | | Age group (years) | | | | | Total | Age group (years) | | | | | Total | Age group (years) | | | | | | Total |
| | | | <30 | 30-39 | 40-49 | 50-59 | 60-69 | | <30 | 30-39 | 40-49 | 50-59 | 60-69 | | <30 | 30-39 | 40-49 | 50-59 | 60-69 | 70-79 | |
| Probands with *BRCA1* PVs | 1st-degree | Breast | 0 | 27 | 7 | 3 | 0 | 37 | 0 | 0 | 2 | 0 | 0 | 2 | 2 | 6 | 14 | 12 | 4 | 0 | 38 |
| | | Ovary | 0 | 4 | 3 | 0 | 0 | 7 | 0 | 0 | 0 | 0 | 0 | 0 | 0 | 3 | 4 | 5 | 4 | 0 | 16 |
| | 2nd-degree | Breast | 4 | 17 | 5 | 0 | 0 | 26 | 0 | 1 | 1 | 0 | 0 | 2 | 1 | 5 | 9 | 3 | 1 | 0 | 19 |
| | | Ovary | 0 | 6 | 1 | 0 | 0 | 7 | 0 | 0 | 1 | 0 | 0 | 1 | 0 | 2 | 5 | 1 | 0 | 0 | 8 |
| Probands with *BRCA2* PVs | 1st-degree | Breast | 1 | 9 | 1 | 1 | 0 | 12 | 0 | 1 | 0 | 0 | 0 | 1 | 0 | 7 | 25 | 34 | 15 | 0 | 81 |
| | | Ovary | 0 | 0 | 0 | 1 | 0 | 1 | 0 | 1 | 0 | 0 | 0 | 1 | 0 | 1 | 4 | 8 | 3 | 0 | 16 |
| | 2nd-degree | Breast | 6 | 11 | 3 | 1 | 0 | 21 | 0 | 1 | 0 | 0 | 0 | 1 | 1 | 11 | 9 | 7 | 1 | 0 | 29 |
| | | Ovary | 0 | 1 | 0 | 0 | 0 | 1 | 1 | 1 | 0 | 0 | 0 | 2 | 0 | 2 | 1 | 1 | 1 | 0 | 5 |
| Probands with *PALB2* PVs | 1st-degree | Breast | 0 | 3 | 1 | 2 | 0 | 6 | 0 | 0 | 0 | 0 | 0 | 0 | 0 | 0 | 3 | 8 | 6 | 0 | 17 |
| | | Ovary | 0 | 0 | 0 | 0 | 0 | 0 | 0 | 0 | 0 | 0 | 0 | 0 | 0 | 0 | 1 | 1 | 0 | 0 | 2 |
| | 2nd-degree | Breast | 3 | 2 | 1 | 2 | 0 | 8 | 0 | 1 | 0 | 0 | 0 | 1 | 0 | 0 | 3 | 0 | 1 | 0 | 4 |
| | | Ovary | 0 | 0 | 0 | 1 | 0 | 1 | 0 | 0 | 0 | 0 | 0 | 0 | 0 | 0 | 1 | 0 | 0 | 0 | 1 |
| Probands with *CHEK2* PVs | 1st-degree | Breast | 0 | 1 | 0 | 1 | 0 | 2 | 0 | 0 | 2 | 0 | 0 | 2 | 1 | 0 | 9 | 22 | 8 | 0 | 40 |
| | | Ovary | 0 | 0 | 0 | 0 | 0 | 0 | 0 | 0 | 0 | 0 | 0 | 0 | 0 | 0 | 0 | 2 | 2 | 0 | 4 |
| | 2nd-degree | Breast | 2 | 4 | 0 | 0 | 0 | 6 | 0 | 0 | 0 | 0 | 0 | 0 | 1 | 1 | 5 | 6 | 3 | 0 | 16 |
| | | Ovary | 1 | 0 | 1 | 0 | 0 | 2 | 0 | 0 | 0 | 0 | 0 | 0 | 0 | 0 | 1 | 1 | 1 | 0 | 3 |
| Probands with *ATM* PVs | 1st-degree | Breast | 0 | 0 | 0 | 0 | 0 | 0 | 0 | 0 | 0 | 0 | 0 | 0 | 0 | 0 | 7 | 13 | 7 | 0 | 27 |
| | | Ovary | 0 | 0 | 0 | 0 | 0 | 0 | 0 | 0 | 0 | 0 | 0 | 0 | 0 | 0 | 1 | 2 | 3 | 0 | 6 |
| | 2nd-degree | Breast | 0 | 1 | 2 | 0 | 0 | 3 | 0 | 1 | 0 | 0 | 0 | 1 | 0 | 0 | 6 | 0 | 3 | 0 | 9 |
| | | Ovary | 0 | 0 | 0 | 0 | 0 | 0 | 0 | 0 | 0 | 0 | 0 | 0 | 0 | 0 | 0 | 0 | 1 | 0 | 1 |
| | | Breast | 0 | 2 | 0 | 1 | 0 | 3 | 0 | 0 | 0 | 0 | 0 | 0 | 1 | 0 | 3 | 0 | 0 | 0 | 4 |

| | | | | | | | | | | | | | | | | | | | | | | | |
|---|---|---|---|---|---|---|---|---|---|---|---|---|---|---|---|---|---|---|---|---|---|---|---|
| Probands with *TP53* PVs | 1st-degree | Ovary | 1 | 0 | 0 | 0 | 0 | 1 | | 0 | 0 | 0 | 0 | 0 | 0 | | 0 | 0 | 0 | 0 | 0 | 0 | 0 |
| | 2nd-degree | Breast | 0 | 9 | 0 | 1 | 0 | 10 | | 0 | 0 | 0 | 0 | 0 | 0 | | 0 | 0 | 1 | 0 | 0 | 0 | 1 |
| | | Ovary | 0 | 0 | 0 | 0 | 0 | 0 | | 0 | 0 | 0 | 0 | 0 | 0 | | 0 | 0 | 0 | 0 | 0 | 0 | 0 |
| Probands without PVs | 1st-degree | Breast | 8 | 63 | 39 | 45 | 4 | 159 | | 2 | 16 | 17 | 14 | 8 | 57 | | 3 | 64 | 353 | 717 | 676 | 1 | 1814 |
| | | Ovary | 0 | 10 | 6 | 5 | 1 | 22 | | 0 | 7 | 6 | 4 | 0 | 17 | | 1 | 7 | 44 | 87 | 62 | 0 | 201 |
| | 2nd-degree | Breast | 15 | 184 | 97 | 94 | 12 | 402 | | 10 | 92 | 48 | 36 | 9 | 195 | | 1 | 65 | 217 | 301 | 169 | 1 | 754 |
| | | Ovary | 1 | 16 | 6 | 8 | 0 | 31 | | 3 | 7 | 5 | 2 | 1 | 18 | | 0 | 8 | 23 | 28 | 11 | 0 | 70 |
| Probands untested | 1st-degree | Breast | 1 | 11 | 3 | 0 | 2 | 17 | | 0 | 3 | 1 | 0 | 1 | 5 | | 1 | 25 | 91 | 137 | 83 | 0 | 337 |
| | | Ovary | 0 | 1 | 0 | 0 | 0 | 1 | | 0 | 0 | 0 | 0 | 0 | 0 | | 1 | 4 | 12 | 13 | 7 | 0 | 37 |
| | 2nd-degree | Breast | 2 | 21 | 2 | 4 | 0 | 29 | | 2 | 7 | 3 | 4 | 5 | 21 | | 2 | 20 | 33 | 45 | 22 | 0 | 122 |
| | | Ovary | 1 | 2 | 0 | 0 | 0 | 3 | | 0 | 2 | 0 | 0 | 1 | 3 | | 0 | 0 | 7 | 1 | 2 | 0 | 10 |

**Table S5 Models including BRCA1, BRCA2, PALB2, CHEK2, ATM, a sixth hypothetical gene and a polygenic component**

| Parameter | Polygenic component only | ***BRCA1, BRCA2*** **and a polygenic component** | ***BRCA1, BRCA2, PALB2, CHEK2, ATM*** **and a polygenic component** | With a sixth hypothetical gene | | | |
|---|---|---|---|---|---|---|---|
| | | | | Dominant inheritance model | Recessive inheritance model | General inheritance model | Dominant inheritance model with an age-constant $\sigma_P^2(t)$ |
| $\sigma_P^2(t)$ (95% CI) [a] | $\alpha$ = 5.442 (95% CI: 4.447, 6.438), $\beta$ = -0.062 (95% CI: -0.079, -0.046) | $\alpha$ = 2.934 (95% CI: 1.854, 4.015), $\beta$ = -0.024 (95% CI: -0.043, -0.005) | $\alpha$ = 2.814 (95% CI: 1.698, 3.929), $\beta$ = -0.023 (95% CI: -0.042, -0.003) | $\alpha$ = 2.303 (95% CI: 1.133, 3.472), $\beta$ = -0.014 (95% CI: -0.035, 0.007) | $\alpha$ = 1.397 (95% CI: 0.257, 2.537), $\beta$ = -0.002 (95% CI: -0.022, 0.018) | $\alpha$ = 2.291 (95% CI: 1.121, 3.460), $\beta$ = -0.014 (95% CI: -0.035, 0.007) | 1.530 (1.373, 1.696) |
| PV allele frequency (95% CI) | | | | | | | |
| *BRCA1* | N/A | 0.082% (0.071%, 0.094%) | 0.081% (0.070%, 0.093%) | 0.080% (0.069%, 0.092%) | 0.080% (0.069%, 0.093%) | 0.080% (0.069%, 0.092%) | 0.080% (0.069%, 0.092%) |
| *BRCA2* | N/A | 0.142% (0.126%, 0.159%) | 0.141% (0.126%, 0.159%) | 0.141% (0.126%, 0.159%) | 0.141% (0.126%, 0.158%) | 0.141% (0.126%, 0.159%) | 0.141% (0.126%, 0.159%) |
| *PALB2* | N/A | N/A | 0.060% (0.049%, 0.073%) | 0.060% (0.049%, 0.073%) | 0.060% (0.049%, 0.073%) | 0.060% (0.049%, 0.073%) | 0.060% (0.049%, 0.073%) |
| *CHEK2* | N/A | N/A | 0.385% (0.338%, 0.438%) | 0.385% (0.338%, 0.439%) | 0.385% (0.338%, 0.438%) | 0.385% (0.338%, 0.439%) | 0.385% (0.338%, 0.438%) |
| *ATM* | N/A | N/A | 0.167% (0.139%, 0.200%) | 0.167% (0.139%, 0.200%) | 0.167% (0.139%, 0.200%) | 0.167% (0.139%, 0.200%) | 0.167% (0.139%, 0.200%) |
| Hypothetical gene | N/A | N/A | N/A | 0.002% (0.001%, 0.006%) | 10.6% (0.5%, 21.1%) | 0.003% (0.001%, 0.006%) | 0.003% (0.001%, 0.008%) |
| RR of hypothetical gene (95% CI) | | | | | | | |
| Heterozygote | N/A | N/A | N/A | 415.87 (283.55, 609.93) | 1 | 398.63 (168.71, 941.89) | 340.15 (143.03, 808.94) |
| Homozygote | N/A | N/A | N/A | 415.87 (283.55, 609.93) | 11.61 (4.25, 31.71) | 36641.33 (0, $1.3 \times 10^{16}$) | 340.15 (143.03, 808.94) |
| Log-likelihood | -48094.17 | -46507.53 | -35414.41 | -35406.04 | -35410.62 | -35406.05 | -35406.76 |
| Number of parameters estimated | 2 | 4 | 7 | 9 | 9 | 10 | 8 |
| Akaike Information Criterion | 96192.34 | 93031.06 | 70842.82 | 70830.08 | 70839.24 | 70832.10 | 70829.52 |
| P [b] | N/A | N/A | N/A | $2.3 \times 10^{-4}$ | 0.02 | $8.1 \times 10^{-4}$ | $9.2 \times 10^{-5}$ |
| Best fitting model | | | | | | | Yes |

[a] $\sigma_P^2(t) = \alpha + \beta \times \text{age}$

[b] From the likelihood ratio test of comparing with the model including *BRCA1*, *BRCA2*, *PALB2*, *CHEK2*, *ATM* and an age-dependent $\sigma_P^2(t)$

**Table S6 Age-specific polygenic variance after fitting major genes and age-specific proportion of breast cancer familial variance explained by major genes**

| Age group (years) | $\sigma_P^2(t)$ (95% CI) [a] | | | | | Proportion of breast cancer familial variance explained by major genes [d] | | | |
|---|---|---|---|---|---|---|---|---|---|
| | Only a polygenic component fitted [b] | *BRCA1, BRCA2* fitted [b] | *BRCA1, BRCA2, PALB2, CHEK2, ATM* fitted [b] | *BRCA1, BRCA2, PALB2, CHEK2, ATM, TP53* fitted [b] | *BRCA1, BRCA2, PALB2, CHEK2, ATM, TP53*, a seventh hypothetical gene fitted [c] | *BRCA1, BRCA2* | *BRCA1, BRCA2, PALB2, CHEK2, ATM* | *TP53* | The seventh hypothetical gene |
| 20-29 | 3.869 (3.274, 4.465) | 2.340 (1.723, 2.959) | 2.245 (1.609, 2.883) | 2.108 (1.472, 2.745) | 1.272 (0.944, 1.649) | 39.52% | 41.98% | 3.54% | 21.59% |
| 30-39 | 3.240 (2.805, 3.679) | 2.102 (1.670, 2.538) | 2.017 (1.572, 2.466) | 1.920 (1.474, 2.370) | 1.272 (0.944, 1.649) | 35.12% | 37.74% | 2.99% | 20.00% |
| 40-49 | 2.611 (2.611, 2.902) | 1.864 (1.599, 2.131) | 1.790 (1.518, 2.063) | 1.733 (1.460, 2.008) | 1.272 (0.944, 1.649) | 28.60% | 31.46% | 2.17% | 17.65% |
| 50-59 | 1.982 (1.803, 2.162) | 1.626 (1.462, 1.789) | 1.562 (1.398, 1.724) | 1.546 (1.380, 1.709) | 1.272 (0.944, 1.649) | 17.95% | 21.19% | 0.83% | 13.79% |
| 60-69 | 1.353 (1.164, 1.543) | 1.389 (1.152, 1.626) | 1.334 (1.096, 1.573) | 1.358 (1.121, 1.596) | 1.272 (0.944, 1.649) | 0% | 1.37% | 0% | 6.35% |
| 70-79 | 0.724 (0.417, 1.032) | 1.151 (0.753, 1.550) | 1.107 (0.701, 1.514) | 1.171 (0.768, 1.575) | 1.272 (0.944, 1.649) | 0% | 0% | 0% | 0% |

[a] For each age group, the variance was assumed to be the variance at the middle point age

[b] From the model in which $\sigma_P^2(t)$ was linearly decreased with age

[c] From the model in which $\sigma_P^2(t)$ was independent with age

[d] For a gene, the age-specific proportion explained by the gene was calculated as the age-specific difference in $\sigma_P^2(t)$ between the model without that gene and the model with that gene divided by the age-specific total breast cancer variance (i.e., column 2). The proportion explained by *BRCA1* and *BRCA2* was calculated as (column 2 – column 3)/column 2, the proportion explained by *BRCA1, BRCA2, PALB2, CHEK2* and *ATM* was calculated as (column 2 – column 4)/column 2, the proportion explained by *TP53* was calculated as (column 4 – column 5)/column 2, the proportion explained by the seventh hypothetical gene was calculated as (column 5 – column 6)/column 2. Where the proportion explained by a gene was negative in older ages, the proportion was assumed to be zero.

**Table S7 Families with the largest change in log-likelihood in favour of the best fitting dominant inheritance model of the hypothetical gene after fitting BRCA1, BRCA2, PALB2, CHEK2, ATM and a polygenic component**

| **Family ID** | **Change in log-likelihood [a]** | **Age at breast cancer diagnosis (years)** | | | | | **PV in other genes not considered in the best fitting dominant inheritance model of the hypothetical gene** |
|---|---|---|---|---|---|---|---|
| | | **Proband** | **Mother** | **Sisters** | **Aunts** | **Grandmothers** | |
| Family 1 | 5.895 | 36 | | 30, 36 | 27 (paternal), 28 (paternal) | 35 | *TP53* |
| Family 2 | 2.983 | 29 | 34 | 34 | | | |
| Family 3 | 1.710 | 39 | | | 27 (paternal), 32(paternal) | | *TP53* |
| Family 4 | 1.477 | 39 | 29 | 24 | | | |
| Family 5 | 1.123 | 24 | 29 | | | | *TP53* |
| Family 6 | 0.885 | 40 | | 20, 23 | | | |
| Family 7 | 0.628 | 27 | 35 | | | | |
| Family 8 | 0.497 | 29 | 34 | | | | |
| Family 9 | 0.374 | 36 | | 33 | | | |
| Family 10 | 0.328 | 41 | 36 | | 35 | | |

[a] The log-likelihood of the best fitting dominant inheritance model of the hypothetic gene minus the log-likelihood of the model including *BRCA1*, *BRCA2*, *PALB2*, *CHEK2*, *ATM* and an age-constant $\sigma_P^2(t)$

**Table S8 Models including BRCA1, BRCA2, PALB2, CHEK2, ATM, TP53 and a polygenic component**

| Parameter | Age-constant *TP53* RR [a] | *TP53* RR varied by every 10 years | *TP53* log-RR as a linear function of age [b] | *TP53* log-RR as a piecewise function of age [c] | *TP53* log-RR as a linear function of age in age 20-49 years and constant in age >49 years, with an age-dependent $\sigma_P^2(t)$ [d] | *TP53* log-RR as a linear function of age in age 20-49 years and constant in age >49 years, with an age-constant $\sigma_P^2(t)$ [e] |
|---|---|---|---|---|---|---|
| $\sigma_P^2(t)$ (95% CI) [f] | α = 2.662 (95% CI: 1.593, 3.731), β = -0.020 (95% CI: -0.039, -0.001) | α = 2.585 (95% CI: 1.424, 3.746), β = -0.019 (95% CI: -0.039, 0.002) | α = 2.600 (95% CI: 1.440, 3.761), β = -0.019 (95% CI: -0.040, 0.001) | α = 2.565 (95% CI: 1.445, 3.686), β = -0.019 (95% CI: -0.038, 0.001) | α = 2.576 (95% CI: 1.473, 3.679), β = -0.019 (95% CI: -0.038, 0.001) | 1.546 (1.389, 1.711) |
| PV allele frequency (95% CI) | | | | | | |
| *BRCA1* | 0.080% (0.069%, 0.092%) | 0.080% (0.069%, 0.092%) | 0.080% (0.069%, 0.092%) | 0.080% (0.069%, 0.092%) | 0.080% (0.069%, 0.092%) | 0.080% (0.069%, 0.092%) |
| *BRCA2* | 0.141% (0.126%, 0.158%) | 0.141% (0.126%, 0.158%) | 0.141% (0.126%, 0.158%) | 0.141% (0.126%, 0.158%) | 0.141% (0.126%, 0.158%) | 0.141% (0.126%, 0.158%) |
| *PALB2* | 0.060% (0.049%, 0.073%) | 0.060% (0.049%, 0.073%) | 0.060% (0.049%, 0.073%) | 0.060% (0.049%, 0.073%) | 0.060% (0.049%, 0.073%) | 0.060% (0.049%, 0.073%) |
| *CHEK2* | 0.385% (0.338%, 0.439%) | 0.385% (0.338%, 0.438%) | 0.385% (0.338%, 0.438%) | 0.385% (0.338%, 0.438%) | 0.385% (0.338%, 0.438%) | 0.385% (0.338%, 0.438%) |
| *ATM* | 0.167% (0.139%, 0.200%) | 0.167% (0.139%, 0.200%) | 0.167% (0.139%, 0.200%) | 0.167% (0.139%, 0.200%) | 0.167% (0.139%, 0.200%) | 0.167% (0.139%, 0.200%) |
| *TP53* | 0.007% (0.005%, 0.011%) | 0.017% (0.009%, 0.034%) | 0.018% (0.008%, 0.041%) | 0.017% (0.009%, 0.045%) | 0.018% (0.009%, 0.036%) | 0.017% (0.009%, 0.034%) |
| RR of *TP53* PVs (95% CI) | | | | | | |
| Age 20-29 years | 36.61 (19.96, 67.17) | 132.61 (55.40, 317.40) | 95.60 (41.19, 222.06) | 170.60 (70.65, 412.55) | 135.75 (61.60, 298.58) | 144.17 (66.24, 311.66) |
| Age 30-39 years | 36.61 (19.96, 67.17) | 30.26 (13.34, 68.65) | 29.83 (13.01, 67.93) | 24.03 (9.74, 59.30) | 30.59 (15.06, 62.38) | 32.13 (16.08, 64.43) |
| Age 40-49 years | 36.61 (19.96, 67.17) | 7.25 (3.07, 17.14) | 9.31 (3.89, 25.52) | 6.92 (2.40, 19.77) | 6.89 (2.48, 19.12) | 7.16 (2.66, 19.24) |
| Age 50-59 years | 36.61 (19.96, 67.17) | 3.07 (0.28, 33.04) | 2.90 (0.79, 10.81) | 2.56 (0.65, 10.11) | 2.95 (1.09, 7.95) | 3.08 (1.16, 8.17) |
| Age 60-69 years | 36.61 (19.96, 67.17) | 3.20 (0.85, 12.04) | 0.91 (0.17, 4.86) | 2.91 (0.69, 12.18) | 2.95 (1.09, 7.95) | 3.08 (1.16, 8.17) |
| Age 70-79 years | 36.61 (19.96, 67.17) | 3.55 (0, 3218679) | 0.28 (0.04, 2.24) | 4.28 (0.25, 70.11) | 2.95 (1.09, 7.95) | 3.08 (1.16, 8.17) |
| Log-likelihood | -35656.36 | 35634.98 | -35638.89 | -35635.99 | -35635.77 | -35637.37 |

| | | | | | | |
|---|---|---|---|---|---|---|
| Number of parameters estimated | 9 | 14 | 10 | 15 | 11 | 10 |
| Akaike Information Criterion | 71330.72 | 71297.96 | 71297.78 | 71298.86 | 71293.54 | 71294.74 |
| P [g] | N/A | $4.1 \times 10^{-8}$ | $3.4 \times 10^{-9}$ | $7.9 \times 10^{-8}$ | $1.1 \times 10^{-9}$ | $7.2 \times 10^{-10}$ |
| Best fitting model | | | | | | Yes |

[a] $P<10^{-15}$ from comparing the model with its equivalent nested model including five major genes and an age-dependent polygenic component. Note that, the log-likelihood of the model including five major genes and an age-decreasing polygenic component in Table S4 is not directly comparable with the log-likelihood of the model additionally including *TP53*, because the two models included different numbers of variables. By fixing the *TP53* allele frequency to be zero and the *TP53* RR to be 1 in the model additionally including *TP53*, the model with an age-dependent $\sigma_P^2(t)$ (essentially the same as the model including five major genes and an age-dependent $\sigma_P^2(t)$) had a log-likelihood of -36210.80 and an AIC of 72435.60.

[b] Log-RR = $\alpha + \beta \times$ (age – 20) in age 20-79 years, where $\alpha$ = 5.22 (95% CI: 4.32, 6.12), $\beta$ = -0.12 (95% CI: -0.16, -0.08)

[c] Log-RR = $\alpha + \beta_1 \times$ (age – 20) in age 20-29 years, $\alpha + 10 \times \beta_1 + \beta_2 \times$ (age – 30) in age 30-39 years, $\alpha + 10 \times (\beta_1 + \beta_2) + \beta_3 \times$ (age – 40) in age 40-49 years, $\alpha + 10 \times (\beta_1 + \beta_2 + \beta_3) + \beta_4 \times$ (age – 50) in age 50-59 years, $\alpha + 10 \times (\beta_1 + \beta_2 + \beta_3 + \beta_4) + \beta_5 \times$ (age – 60) in age 60-69 years, and $\alpha + 10 \times (\beta_1 + \beta_2 + \beta_3 + \beta_4 + \beta_5) + \beta_6 \times$ (age – 70) in age 70-79 years, where $\alpha$ = 6.33 (95% CI: 4.91, 7.75), $\beta_1$ = -0.23 (95% CI: -0.40, -0.05), $\beta_2$ = -0.17 (95% CI: -0.30, -0.04), $\beta_3$ = -0.08 (95% CI: -0.22, 0.06), $\beta_4$ = -0.12 (95% CI: -0.32, 0.08), $\beta_5$ = 0.15 (95% CI: -0.12, 0.41), $\beta_6$ = -0.07 (95% CI: -0.67, 0.53)

[d] Log-RR = $\alpha + \beta \times$ (age – 20) in age 20-49 years, where $\alpha$ = 5.66 (95% CI: 4.69, 6.62), $\beta$ = -0.15 (95% CI: -0.21, -0.09)

[e] Log-RR = $\alpha + \beta \times$ (age – 20) in age 20-49 years, where $\alpha$ = 5.72 (95% CI: 4.78, 6.66), $\beta$ = -0.15 (95% CI: -0.21, -0.09)

[f] $\sigma_P^2(t) = \alpha + \beta \times$ age

[g] From the likelihood ratio test of comparing with the model including an age-constant *TP53* RR

**Table S9 Families with the largest change in log-likelihood in favour of the best fitting recessive inheritance model of the hypothetical gene after fitting BRCA1, BRCA2, PALB2, CHEK2, ATM, TP53 and a polygenic component**

| Family ID | Change in log likelihood [a] | Age at breast cancer diagnosis (years) | | | | |
|---|---|---|---|---|---|---|
| | | Proband | Mother | Sisters | Aunts | Grandmothers |
| Family 1 | 0.676 | 29 | 34 | 34 | | |
| Family 2 | 0.519 | 50 | 32 | 39, 45 | | |
| Family 3 | 0.486 | 31 | 46 | 37 | | |
| Family 4 | 0.485 | 38 | | 42, 46 | 66 (maternal) | |
| Family 5 | 0.484 | 43 | | 30, 30 | | |
| Family 6 | 0.472 | 62 | 54 | 44, 51, 53 | | 54 (paternal) |
| Family 7 | 0.462 | 47 | | 37, 42 | | |
| Family 8 | 0.437 | 45 | 46 | 38 | | 36 (paternal) |
| Family 9 | 0.362 | 48 | | 45, 50 | | |
| Family 10 | 0.329 | 46 | | 45 | | |

[a] The log-likelihood of the best fitting recessive inheritance model of the hypothetical gene minus the log-likelihood of the model including *BRCA1*, *BRCA2*, *PALB2*, *CHEK2*, *ATM*, *TP53* and an age-constant $\sigma_P^2(t)$

**Table S10 Sensitivity analyses results of assuming the pathogenic variant test sensitivity to be 80% for models including BRCA1, BRCA2, PALB2, CHEK2, ATM, TP53, a polygenic component, with or without a hypothetical gene**

| Parameters | Without the hypothetical gene | With the hypothetical gene | | | |
|---|---|---|---|---|---|
| | Age-constant $\sigma_P^2(t)$ | Dominant inheritance model | Recessive inheritance model [a] | General inheritance model | Recessive inheritance model with an age-dependent $\sigma_P^2(t)$ [b] |
| $\sigma_P^2(t)$ (95% CI) | 1.493 (1.336, 1.658) | 1.486 (1.330, 1.652) | 1.252 (0.950, 1.595) | 1.252 (0.935, 1.615) | $\alpha$ = 0.742 (95% CI: -0.739, 2.223), $\beta$ = 0.009 (95% CI: -0.016, 0.034) |
| PV allele frequency (95% CI) | | | | | |
| *BRCA1* | 0.089% (0.078%, 0.103%) | 0.089% (0.077%, 0.103%) | 0.089% (0.077%, 0.103%) | 0.089% (0.077%, 0.103%) | 0.089% (0.077%, 0.103%) |
| *BRCA2* | 0.157% (0.140%, 0.177%) | 0.157% (0.140%, 0.177%) | 0.157% (0.140%, 0.176%) | 0.157% (0.140%, 0.176%) | 0.157% (0.140%, 0.176%) |
| *PALB2* | 0.067% (0.055%, 0.082%) | 0.067% (0.055%, 0.082%) | 0.067% (0.055%, 0.081%) | 0.067% (0.055%, 0.081%) | 0.067% (0.055%, 0.081%) |
| *CHEK2* | 0.432% (0.379%, 0.492%) | 0.432% (0.379%, 0.492%) | 0.432% (0.379%, 0.492%) | 0.432% (0.379%, 0.492%) | 0.432% (0.379%, 0.492%) |
| *ATM* | 0.187% (0.156%, 0.224%) | 0.187% (0.156%, 0.224%) | 0.187% (0.156%, 0.224%) | 0.187% (0.156%, 0.224%) | 0.187% (0.156%, 0.224%) |
| *TP53* | 0.020% (0.010%, 0.039%) | 0.020% (0.008%, 0.051%) | 0.020% (0.010%, 0.041%) | 0.020% (0.010%, 0.042%) | 0.020% (0.009%, 0.043%) |
| Hypothetical gene | N/A | 0.001% (0.0001%, 0.006%) | 12.6% (5.4%, 21.8%) | 12.6% (5.2%, 22.5%) | 12.7% (6.2%, 19.6%) |
| RR of *TP53* PVs (95% CI) | | | | | |
| Age 20-29 years | 138.06 (64.62, 294.21) | 132.33 (54.36, 320.67) | 138.66 (60.99, 313.92) | 138.66 (59.76, 320.30) | 144.85 (59.03, 353.78) |
| Age 30-39 years | 31.13 (15.55, 62.59) | 30.30 (12.32, 74.73) | 31.32 (15.02, 65.66) | 31.32 (14.55, 67.78) | 32.41 (14.39, 73.28) |
| Age 40-49 years | 7.02 (2.58, 19.12) | 6.94 (2.05, 23.47) | 7.07 (2.57, 19.43) | 7.07 (2.50, 20.02) | 7.25 (2.52, 20.95) |
| Age 50-59 years | 2.99 (1.08, 8.30) | 2.93 (0.56, 15.37) | 3.03 (0.98, 9.37) | 3.03 (0.89, 10.33) | 3.12 (0.87, 11.21) |
| Age 60-69 years | 2.99 (1.08, 8.30) | 2.93 (0.56, 15.37) | 3.03 (0.98, 9.37) | 3.03 (0.89, 10.33) | 3.12 (0.87, 11.21) |
| Age 70-79 years | 2.99 (1.08, 8.30) | 2.93 (0.56, 15.37) | 3.03 (0.98, 9.37) | 3.03 (0.89, 10.33) | 3.12 (0.87, 11.21) |

| | | | | | |
|---|---|---|---|---|---|
| RR of the hypothetical gene (95% CI) | | | | | |
| Heterozygote | N/A | 367.24 (308.71, 436.87) | 1 | 1 | 1 |
| Homozygote | N/A | 367.24 (308.71, 436.87) | 9.69 (4.09, 22.98) | 9.69 (4.06, 23.12) | 10.59 (5.01, 22.38) |
| Log-likelihood | -35636.08 | -35635.20 | -35631.95 | -35631.95 | -35631.74 |
| Number of parameters estimated | 10 | 12 | 12 | 13 | 13 |
| Akaike Information Criterion | 71292.16 | 71294.4 | 71287.90 | 71289.90 | 71289.48 |
| P [c] | N/A | 0.41 | 0.02 | 0.04 | 0.03 |
| Best fitting model | | | Yes | | |

[a] Log-RR = $\alpha + \beta \times (\text{age} - 20)$ in age 20-49 years, where $\alpha = 5.68$ (95% CI: 4.69, 6.66), $\beta = -0.15$ (95% CI: -0.20, -0.09)

[b] $\sigma_P^2(t) = \alpha + \beta \times \text{age}$

[c] From the likelihood ratio test of comparing with the model including *BRCA1*, *BRCA2*, *PALB2*, *CHEK2*, *ATM*, *TP53* and an age-constant $\sigma_P^2(t)$

**Supplemental Acknowledgements**

The ABCFR was supported in Australia by the National Health and Medical Research Council (NHMRC), the New South Wales Cancer Council, the Victorian Health Promotion Foundation, the Victorian Breast Cancer Research Consortium, Cancer Australia, and the National Breast Cancer Foundation. The six sites of the Breast Cancer Family Registry (BCFR) were supported by grant UM1 CA164920 from the U.S. National Cancer Institute. The content of this manuscript does not necessarily reflect the views or policies of the National Cancer Institute or any of the collaborating centres in the BCFR, nor does mention of trade names, commercial products, or organizations imply endorsement by the U.S. Government or the BCFR. Sequencing of the ABCFR samples was supported by an NHMRC Program grant (APP1074383), The National Breast Cancer Foundation (BRA-STRAP; NT-15-016), NHMRC European Union Collaborative Research Grant (APP1101400) and Monash University, Melbourne, Australia. SEARCH was funded by Cancer Research UK (C490/A16561) and the NIHR Biomedical Research Centre at the University of Cambridge and the University of Cambridge has received salary support for P.D.P.P. from the NHS in the East of England through the Clinical Academic Reserve. Sequencing of the SEARCH samples was funded by European Union's Horizon 2020 Research and Innovation Programme (BRIDGES: grant number 634935).
