# Supplementary material for: Segregation analysis of 17,425 population-based breast cancer families: Evidence for genetic susceptibility and risk prediction
Source: Am J Hum Genet. 2022 Oct 6;109(10):1777–88. doi: 10.1016/j.ajhg.2022.09.006 (PMC9606477; doi:10.1016/j.ajhg.2022.09.006)
Supplement: Document S2. Article plus supplemental information [file mmc2.pdf]

Segregation analysis of 17,425 population-based breast cancer families: Evidence for genetic susceptibility and risk prediction

Authors

Shuai Li, Robert J. MacInnis, Andrew Lee, ...,
Douglas F. Easton, John L. Hopper,
Antonis C. Antoniou

Correspondence

shuai.li@unimelb.edu.au

A large population-based family study, with gene-panel sequencing data, investigated the genetic models that explain the residual breast cancer familial aggregation after considering known susceptibility genes. The results may have implications for strategies to identify new breast cancer-susceptibility genes and improve disease-risk prediction, especially at a young age.

Li et al., 2022, The American Journal of Human Genetics *109*, 1777–1788
October 6, 2022 © 2022 The Author(s).

https://doi.org/10.1016/j.ajhg.2022.09.006

Segregation analysis of 17,425 population-based breast cancer families: Evidence for genetic susceptibility and risk prediction

Shuai Li,[1,2,3,4,*] Robert J. MacInnis,[1,5] Andrew Lee,[2] Tu Nguyen-Dumont,[3,6] Leila Dorling,[2] Sara Carvalho,[2] Gillian S. Dite,[1,7] Mitul Shah,[8] Craig Luccarini,[8] Qin Wang,[2] Roger L. Milne,[1,3,5] Mark A. Jenkins,[1] Graham G. Giles,[1,3,5] Alison M. Dunning,[8] Paul D.P. Pharoah,[2] Melissa C. Southey,[3,5,6] Douglas F. Easton,[2] John L. Hopper,[1,9] and Antonis C. Antoniou[2,9]

Summary

Rare pathogenic variants in known breast cancer-susceptibility genes and known common susceptibility variants do not fully explain the familial aggregation of breast cancer. To investigate plausible genetic models for the residual familial aggregation, we studied 17,425 families ascertained through population-based probands, 86% of whom were screened for pathogenic variants in *BRCA1*, *BRCA2*, *PALB2*, *CHEK2*, *ATM*, and *TP53* via gene-panel sequencing. We conducted complex segregation analyses and fitted genetic models in which breast cancer incidence depended on the effects of known susceptibility genes and other unidentified major genes and a normally distributed polygenic component. The proportion of familial variance explained by the six genes was 46% at age 20–29 years and decreased steadily with age thereafter. After allowing for these genes, the best fitting model for the residual familial variance included a recessive risk component with a combined genotype frequency of 1.7% (95% CI: 0.3%–5.4%) and a penetrance to age 80 years of 69% (95% CI: 38%–95%) for homozygotes, which may reflect the combined effects of multiple variants acting in a recessive manner, and a polygenic variance of 1.27 (95% CI: 0.94%–1.65), which did not vary with age. The proportion of the residual familial variance explained by the recessive risk component was 40% at age 20–29 years and decreased with age thereafter. The model predicted age-specific familial relative risks consistent with those observed by large epidemiological studies. The findings have implications for strategies to identify new breast cancer-susceptibility genes and improve disease-risk prediction, especially at a young age.

Introduction

There is a substantial familial aggregation of breast cancer (MIM: 114480). The familial relative risk (FRR) of breast cancer for having an affected first-degree relative is on average about 1.8 but is greater the younger the age at cancer diagnosis of the relative(s), the greater the number of affected relatives, and the younger the age of the consultand.[1]

High-risk pathogenic variants (PVs) in the currently known breast cancer-susceptibility genes *BRCA1* (MIM: 113705), *BRCA2* (MIM: 600185), and *PALB2* (MIM: 610355) and intermediate-risk PVs in genes such as *CHEK2* (MIM: 604373) and *ATM* (MIM: 607585)[2–10] on average explain 20%–25% of the familial aggregation of breast cancer and much more at younger ages. A further on average ~20% of the familial aggregation is accounted for by a polygenic risk score (PRS) based on 313 common genetic variants identified by genome-wide association studies (GWASs).[11] PVs in other genes, including *BARD1* (MIM: 601593), *RAD51C* (MIM: 602774), *RAD51D* (MIM: 602954), *TP53* (MIM: 191170), *PTEN* (MIM: 601728), and *NF1* (MIM: 613113), are also associated with breast cancer risk, but to a lesser extent, and account for a small proportion of the familial aggregation.[2] Consequently, a large proportion of the familial aggregation remains unexplained.

Segregation analyses have been used to develop pedigree-based statistical models of breast cancer susceptibility and predict breast cancer risk on the basis of family history, genotype, and other factors.[12–15] For example, the Breast and Ovarian Analysis of Disease Incidence and Carrier Estimation Algorithm (BOADICEA) for estimating a woman's future risk of developing breast cancer[16–20] was originally developed with data on 2,785 families and considered breast cancer familial risk to be determined by the joint effects of *BRCA1* and *BRCA2* PVs and a polygenic component representing the combined multiplicative effects of a large number of unknown genetic variants each making a small contribution to the variation in risk.[12,14,17] The model was subsequently extended to incorporate PVs in *PALB2*, *CHEK2*, and *ATM*, polygenic risk scores, lifestyle- and

[1]Centre for Epidemiology and Biostatistics, Melbourne School of Population and Global Health, The University of Melbourne, 207 Bouverie Street, Carlton, VIC 3053, Australia; [2]Centre for Cancer Genetic Epidemiology, Department of Public Health and Primary Care, University of Cambridge, Cambridge CB1 8RN, UK; [3]Precision Medicine, School of Clinical Sciences at Monash Health, Monash University, Clayton, VIC 3168, Australia; [4]Murdoch Children's Research Institute, Royal Children's Hospital, Parkville, VIC 3051, Australia; [5]Cancer Epidemiology Division, Cancer Council Victoria, Melbourne, VIC 3004, Australia; [6]Department of Clinical Pathology, The University of Melbourne, Parkville, VIC 3051, Australia; [7]Genetic Technologies Ltd., Fitzroy, VIC 3065, Australia; [8]Centre for Cancer Genetic Epidemiology, Department of Oncology, University of Cambridge, Cambridge CB1 8RN, UK
[9]These authors contributed equally
*Correspondence: shuai.li@unimelb.edu.au
https://doi.org/10.1016/j.ajhg.2022.09.006.
© 2022 The Author(s). This is an open access article under the CC BY license (http://creativecommons.org/licenses/by/4.0/).

hormone-related risk factors, and mammographic density[20] and more recently PVs in *BARD1*, *RAD51C*, and *RAD51D*.[21]

It was not previously possible to fit segregation analysis models that included the joint effects of all known breast cancer-susceptibility genes simultaneously because of the lack of data on PVs in some genes. Another challenge has been small sample sizes that limit the statistical power to distinguish different inheritance models. In this study, we conducted the analysis of this kind by using data from families ascertained through large population-based series of affected and unaffected probands for which data on PVs in *BRCA1*, *BRCA2*, *PALB2*, *CHEK2*, *ATM*, and *TP53* were available. Our objectives were (1) to estimate key genetic model parameters simultaneously to further improve the accuracy of existing genetic models and (2) to investigate the genetic models of inheritance that best explain the familial aggregation of breast cancer not accounted for by the known breast cancer-susceptibility genes and polygenic factors.

Material and methods

Study sample

The sample included 17,425 three-generation families ascertained via population-based sampling of breast cancer probands from two studies: 2,712 families from the population-based case-control family study within the Australian Breast Cancer Family Registry (ABCFR) and 14,713 families from the prospective Studies of Epidemiology and Risk Factors in Cancer Heredity (SEARCH) study in the UK.

ABCFR[22–24] includes (1) 1,644 case families, ascertained independently of their family history through a sample of adult women living in the metropolitan areas of Melbourne and Sydney who were diagnosed between 1992 and 1999 (baseline) with a histologically confirmed first primary breast cancer (case probands) before age 70 years and (2) 1,068 control families ascertained through unaffected adult women (control probands) who were sampled at the same time via the Australian electoral rolls and frequency matched to case probands by age. Of these, 858 case families with a proband diagnosed with breast cancer before age 40 years were included in a previous segregation analysis.[13] Case and control probands gave a blood sample and completed the same risk factor questionnaire and family history questionnaire involving the construction of a pedigree covering all known first- and second-degree adult relatives. In addition, each proband was asked to obtain permission from first- and second-degree relatives for their participation, which involved giving a blood sample, completing the same risk factor questionnaire, and providing additional information to complement the pedigree and family history information collected from the proband. This analysis used demographic data and breast and ovarian cancer (MIM: 167000) diagnoses of the probands and all their adult female first- and second-degree relatives identified at baseline. ABCFR was designed to be enriched for breast cancer diagnoses at younger ages: 55% of case probands and 52% of control probands included in this analysis were younger than 40 years at diagnosis and recruitment, respectively. Of the probands, 92% reported having White ethnicity.

SEARCH[25] ascertained families through adult women diagnosed with breast cancer, identified through the Eastern Cancer Registration and Information Centre. Eligible women were those diagnosed between 1991 and 1996 before 55 years of age and recruited between 1996 and 2002 (prevalent cases) together with women diagnosed between 1996 and 2011 before age 70 years (incident cases). Probands were invited to provide a blood sample and complete an epidemiological questionnaire, including family history in all first-degree relatives and grandparents. This analysis used the demographic data and breast and ovarian cancer diagnoses of the probands and all their adult female first-degree relatives and grandmothers collected at baseline. A total of 1,484 families with a proband diagnosed before age 55 years between 1991 and 1996 were included in previous segregation analyses.[12,14] Of the probands included in this analysis, 94% were older than 40 years at diagnosis. Approximately three-quarters of the probands reported their ethnicity, and 99% had White European ancestry.

ABCFR was approved by the Human Research Ethics Committee of the University of Melbourne. SEARCH was approved by the National Research Ethics Service Committee East of England—Cambridge South. All participants provided written consent.

PVs in breast cancer-susceptibility genes

We studied six major cancer-susceptibility genes: *BRCA1*, *BRCA2*, *PALB2*, *CHEK2*, *ATM*, and *TP53*. PVs included predicted protein-truncating variants (PTVs) and an additional subset of rare missense variants (population frequency < 0.001) for *BRCA1*, *BRCA2*, and *TP53*. The risks associated with this subset of missense variants have been shown to be similar to those associated with PTVs,[2] and these were aggregated with the PTVs in the analysis.

In ABCFR, PVs were identified by gene-panel testing for 2,305 probands (85% of all probands) and 770 relatives and by other tests conducted before gene-panel testing was widely available (Table S1) for 2,317 probands and 1,765 relatives; 2,244 probands and 611 relatives tested by both regimes. In total, 88% of families were tested; see Southey et al.[26] for more details about the gene-panel testing and pathogenicity definition. In SEARCH, PVs were identified by gene-panel testing for 12,654 probands (86% of all probands). No relatives were screened; see Dorling et al.[2] for more details about the testing and pathogenicity definition. We effectively applied the American College of Medical Genetics and Genomics (ACMG) and the Association for Molecular Pathology (AMP) criteria to classify variant pathogenicity, as our study used pathogenicity classification reviewed by the Evidence-based Network for the Interpretation of Germline Mutant Alleles (ENIGMA) Expert Panel.

Statistical methods

We conducted complex segregation analysis by using the pedigree analysis software MENDEL following a similar approach to that used in previous studies.[12–14] Each woman was considered to be at risk of breast and ovarian cancer from birth until breast or ovarian cancer diagnosis, baseline interview, death, or age 80 years (whichever occurred first). The incidences of the two cancers were modeled simultaneously and assumed to be independent, conditional on genotype. For a woman i in birth cohort k, from country c and at age t, the incidence of cancer s, $\lambda_i(t, k, c, s)$, was assumed to depend on the genetic factors according to the following model:

$$\lambda_i(t,\ k,\ c,\ s) = \lambda_0(t,\ k, c,\ s)\exp\left(\sum_{\mu=1}^{n}\beta_{MG_\mu}(t,\ s)G_{\mu i} + P_i(t)\right)$$

where $\lambda_0(t, k, c, s)$ is the baseline incidence, $\beta_{MG_\mu}(t,\ s)$ is the age-specific log-relative risk (log-RR) for a major gene that was assumed

to be the same across countries and birth cohorts, n is the number of major genotypes, and $G_{\mu i}$ is an indicator variable that takes the value of 1 if the woman has major genotype μ and 0 otherwise. $P_i(t)$ is the breast cancer age-specific polygenic component that was assumed to be normally distributed with zero mean and variance $\sigma_P^2(t)$, the same across countries and birth cohorts, representing the multiplicative effects of a large number of variants each associated with a small increment in breast cancer risk. No polygenic component was included for ovarian cancer. The polygenic component was approximated by the hypergeometric polygenic model (HPM)[27,28] as

$$P = \frac{R - N}{\sqrt{\frac{N}{2}}}\sigma_P$$

where R has a binomial distribution (2N, $^1/_2$) and N, the number of loci used in the HPM, was 3.

We firstly fitted a model with a polygenic component only, without any major genes. The polygenic variance $\sigma_P^2(t)$ from this model reflects the total breast cancer familial variance under the polygenic susceptibility model.[29] Note that while $\sigma_P^2(t)$ is termed the polygenic variance, it also captures the effects of non-genetic factors contributing to the risks for relatives being correlated and whose existence is suggested by analysis of twin pairs in the Nordic Twin Study.[30] Given that breast cancer FRR decreases with age,[1] we allowed $\sigma_P^2(t)$ to decrease linearly with age.

We then fitted a model equivalent to the BOADICEA (versions 4 and 5) in terms of the number of major genes, which included the effects of PVs in *BRCA1*, *BRCA2*, *PALB2*, *CHEK2*, and *ATM*, as well as the polygenic component. We sequentially extended this model to include the effects of PVs in *TP53*, and a hypothetical major gene for which we investigated different models of inheritance (dominant, recessive, general). We used the decrease in $\sigma_P^2(t)$ as major genes were included to express the proportions of breast cancer familial variance explained by PVs in those genes.

To reduce computational time, we fitted the major gene component by using a single locus comprising $m+1$ alleles, where m is the number of major genes considered in the model. That is, we assumed separate risk alleles representing the presence of a PV in *BRCA1*, *BRCA2*, *PALB2*, *CHEK2*, *ATM*, *TP53*, and the hypothetical gene and a normal allele. We assumed a dominant inheritance for the risk alleles in the order of the seven major genes above; therefore, there was a total of nine possible major genotypes μ: women with *BRCA1* PVs, women with *BRCA2* PVs, women with *PALB2* PVs, women with *CHEK2* PVs, women with *ATM* PVs, women with *TP53* PVs, the hypothetical gene risk allele homozygotes, the hypothetical gene risk allele heterozygotes, and women without PVs. This simplification is unlikely to affect results because women with PVs in more than one major gene are very rare and would not contribute materially to the analysis; from our data, 0.1% of the women were such (Table 1). To compute the baseline incidence $\lambda_0(t, k, c, s)$, we used the method previously described[12] to constrain the overall incidence across all genotypes to agree with the UK and Australian birth-cohort-specific smoothed population incidences used in the BOADICEA.[18]

The age-specific breast and ovarian cancer RRs ($\beta_{MG_\mu}(t, s)$) for *BRCA1* and *BRCA2* PVs and breast cancer RRs for *PALB2*, *CHEK2*, and *ATM* PVs were fixed at estimates from previous studies[5,7,9,17]: log-RRs were functions of age for *BRCA1*, *BRCA2*, *PALB2*, and *CHEK2* PVs (Table S2), and breast cancer RR was 2.8 for *ATM* PVs. Ovarian cancer RRs were assumed to be 1 for *PALB2*, *CHEK2*, and *ATM* PVs, i.e., ovarian cancer incidence depended on *BRCA1* and *BRCA2* PVs only. The risk allele frequencies for all major genes, the age-specific RRs for *TP53* PVs and hypothetical gene risk allele, and the age-specific polygenic variance $\sigma_P^2(t)$ were estimated. The variances of the parameter estimates were obtained by inverting the observed information matrix. To allow for the restricted ranges of the parameter values and provide estimates likely to be more nearly normally distributed, we used transformed values for the parameters in the model: allele frequencies were logit transformed, RRs were log transformed, and $\sigma_P^2(t)$ was square-root transformed. We compared nested models by using the likelihood ratio test and non-nested models by using the Akaike information criterion. All statistical tests were two sided, and results with a $p < 0.05$ were treated as statistically significant.

Because not all PVs would be detected by the test methods used, we included a test sensitivity parameter defined as the probability of detecting a PV if one exists. In ABCFR, the test sensitivity was the weighted sum of the sensitivities of the test methods used (Table S1), and weights were the proportional lengths of the exons screened and taking into account whether multiplex ligation-dependent probe amplification had been conducted to detect large rearrangements and copy-number variations. The test sensitivity was assumed to be 100% for the relatives who had been only tested for their probands' PV. For ABCFR probands, the average test sensitivity ranged from 88% to 90% across all genes. For SEARCH probands, the test sensitivity was assumed to be 90% for all genes. We also conducted a sensitivity analysis by assuming the test sensitivity to be 80% for all genes and all probands.

To adjust for family ascertainment, we computed the likelihood of observing the phenotypes and genotypes of each family conditional on observing the phenotypes of the proband, i.e., their cancer status and age of diagnosis or censoring. This ascertainment is justified because all families were ascertained through population-based sampling of probands, and the family history and genotype data could be assumed not to influence the ascertainment.

We computed age-specific breast cancer FRR predicted by the best fitting model by using the likelihoods from MENDEL.[16] The FRR for a woman at age t with a first-degree relative affected at age t was computed as the ratio of two pedigree-likelihoods:

$$FRR = \frac{P(The\ woman\ affected\ at\ age\ t |\ The\ relative\ affected\ at\ age\ t)}{P(The\ woman\ affected\ at\ age\ t)}$$
$$= \frac{P(The\ woman\ affected\ at\ age\ t,\ The\ relative\ affected\ at\ age\ t)}{P(The\ woman\ affected\ at\ age\ t)P(The\ relative\ affected\ at\ age\ t)}.$$

We computed the FRRs separately for women with an affected mother and women with an affected sister.

For *TP53* PVs and the hypothetical gene risk allele, we estimated the age-specific cumulative risk (penetrance) to age t, $F(t)$, as

$$F(t) = 1 - \exp\left(-\int_0^t \lambda(t)dt\right)$$

where $\lambda(t)$ is the estimated incidence at age t averaged over the polygenic effects, based on the UK population incidence for women born in 1940–1949 (the median birth year of the probands was 1948). The 95% confidence interval (CI) of $F(t)$ was calculated via a parametric bootstrap: a sample of 10,000 draws was taken from the multivariate normal distribution that the maximum likelihood estimates would be expected to follow under asymptotic likelihood theory; for each age, a corresponding sample of 10,000 cumulative risks was calculated with the

Table 1. Number and proportion of probands by study and the diagnosis age and pathogenic variant (PV) status of the proband[a]

Age group (years)	Total	Probands with *BRCA1* PVs	Probands with *BRCA2* PVs	Probands with *PALB2* PVs	Probands with *CHEK2* PVs	Probands with *ATM* PVs	Probands with *TP53* PVs	Probands without PVs	Untested
**ABCFR case families**									
<30	88	7 (8.6%)	6 (7.4%)	1 (1.2%)	2 (2.5%)	2 (2.5%)	3 (3.7%)	60 (74.1%)	7
30–39	810	43 (6.0%)	27 (3.8%)	1 (0.1%)	11 (1.5%)	5 (0.7%)	5 (0.7%)	628 (87.3%)	91
40–49	368	15 (4.3%)	5 (1.4%)	4 (1.2%)	6 (1.7%)	3 (0.9%)	0 (0%)	313 (90.7%)	23
50–59	346	2 (0.6%)	6 (1.8%)	5 (1.5%)	2 (0.6%)	3 (0.9%)	3 (0.9%)	315 (93.8%)	10
60–69	32	0 (0%)	0 (0%)	0 (0%)	0 (0%)	1 (3.4%)	1 (3.4%)	27 (93.1%)	3
Total	1,644	67 (4.4%)	44 (2.9%)	11 (0.7%)	21 (1.4%)	14 (0.9%)	12 (0.8%)	1,343 (88.9%)	134
**ABCFR control families**									
<30	86	0 (0%)	1 (1.7%)	0 (0%)	1 (1.7%)	3 (5.0%)	0 (0%)	55 (91.7%)	26
30–39	468	2 (0.5%)	3 (0.8%)	1 (0.3%)	0 (0%)	1 (0.3%)	0 (0%)	366 (98.4%)	96
40–49	240	2 (0.9%)	1 (0.5%)	0 (0%)	2 (0.9%)	0 (0%)	0 (0%)	206 (97.6%)	29
50–59	218	0 (0%)	0 (0%)	0 (0%)	1 (0.5%)	2 (1.1%)	1 (0.5%)	179 (97.8%)	35
60–69	56	0 (0%)	0 (0%)	0 (0%)	0 (0%)	0 (0%)	0 (0%)	42 (100%)	14
Total	1,068	4 (0.5%)	6 (0.6%)	1 (0.1%)	4 (0.5%)	6 (0.7%)	1 (0.1%)	848 (97.7%)	200
**SEARCH**									
<30	60	4 (8.9%)	2 (4.4%)	1 (2.2%)	3 (6.7%)	0 (0%)	1 (2.2%)	35 (77.8%)	15
30–39	878	19 (2.8%)	29 (4.2%)	1 (0.1%)	15 (2.2%)	1 (0.1%)	5 (0.7%)	614 (89.8%)	194
40–49	3,519	40 (1.4%)	72 (2.5%)	23 (0.8%)	53 (1.8%)	21 (0.7%)	6 (0.2%)	2,665 (92.6%)	642
50–59	5,694	27 (0.5%)	79 (1.6%)	39 (0.8%)	83 (1.7%)	37 (0.8%)	1 (0.02%)	4,648 (94.6%)	782
60–69	4,558	10 (0.2%)	41 (1.0%)	18 (0.4%)	49 (1.2%)	31 (0.8%)	1 (0.02%)	3,983 (96.4%)	426
70–79	4	0 (0%)	0 (0%)	0 (0%)	0 (0%)	0 (0%)	0 (0%)	4 (100%)	0
Total	14,713	100 (0.8%)	223 (1.8%)	82 (0.6%)	203 (1.6%)	90 (0.7%)	14 (0.1%)	11,949 (94.4%)	2,059

[a]Ten Probands had a PV in two genes: ABCFR case families—one proband (30–39 years) in *BRCA1* and *BRCA2* and one proband (40–49 years) in *BRCA1* and *CHEK2*; ABCFR control families—one proband (30–39 yeas) in *BRCA2* and *ATM*; SEARCH—two probands (40–49 years, 60–69 years) in *BRCA1* and *CHEK2*, one proband (50–59 years) in *BRCA2* and *PALB2*, and four probands (one <30 years, two 40–49 years, and one 50–59 years) in *BRCA2* and *CHEK2*. Within each age group, proportions (percentages in the parentheses) were calculated within the tested probands only, i.e., untested probands were excluded.

formula above, and the 2.5th and 97.5th percentiles of this distribution were taken to be the 95% CI limits.

Results

A total of 15,032 (86%) probands were screened, and 892 (5.9%) were found to carry a PV in *BRCA1*, *BRCA2*, *PALB2*, *CHEK2*, *ATM*, or *TP53* (Table 1). Of the 1,924 ABCFR relatives tested, 142 from 88 families were found to carry a PV (Table S3), and 164 from 86 families did not carry the PV found in their affected probands. 2,449 (14.1%) and 1,521 (8.7%) probands had a family history of breast cancer in first-degree relatives and second-degree relatives, respectively; the corresponding numbers for a family history of ovarian cancer were 328 (1.9%) and 165 (0.9%), respectively (Table S4).

When a polygenic-component-only model was fitted, $\sigma_P^2(t)$ was 3.86 (95% CI: 3.27, 4.47) at age 20–29 years (calculated with the middle point, i.e., 25 years; the same for the age ranges below), decreasing to 0.72 (95% CI: 0.42, 1.03) at age 70–79 years (Table S5). After additionally fitting *BRCA1*, *BRCA2*, *PALB2*, *CHEK2*, and *ATM*, $\sigma_P^2(t)$ was 2.25 (95% CI: 1.61, 2.88) at age 20–29 years, decreasing to 1.11 (95% CI: 0.70, 1.51) at age 70–79 years. The proportion of the total breast cancer familial variance attributed to these five major genes was therefore 42% at 20–29 years and decreased steadily with age thereafter (Figure 1; Table S6).

We then additionally fitted a sixth hypothetical major gene (Table S5). All three inheritance models (dominant, recessive, and general) that included the hypothetical gene gave a better fit than their equivalent nested models without the additional gene (all $p < 0.03$). The dominant inheritance model gave the best fit, and the general inheritance model essentially converged to the dominant inheritance model. Under the dominant inheritance model, the hypothetical gene had a risk allele frequency of 0.003% (95% CI: 0.001%, 0.008%) and an RR of 340 (95% CI: 140, 810); $\sigma_P^2(t)$ was 1.53 (95% CI: 1.37, 1.70), and there was no evidence that $\sigma_P^2(t)$ depended on age ($p = 0.2$).

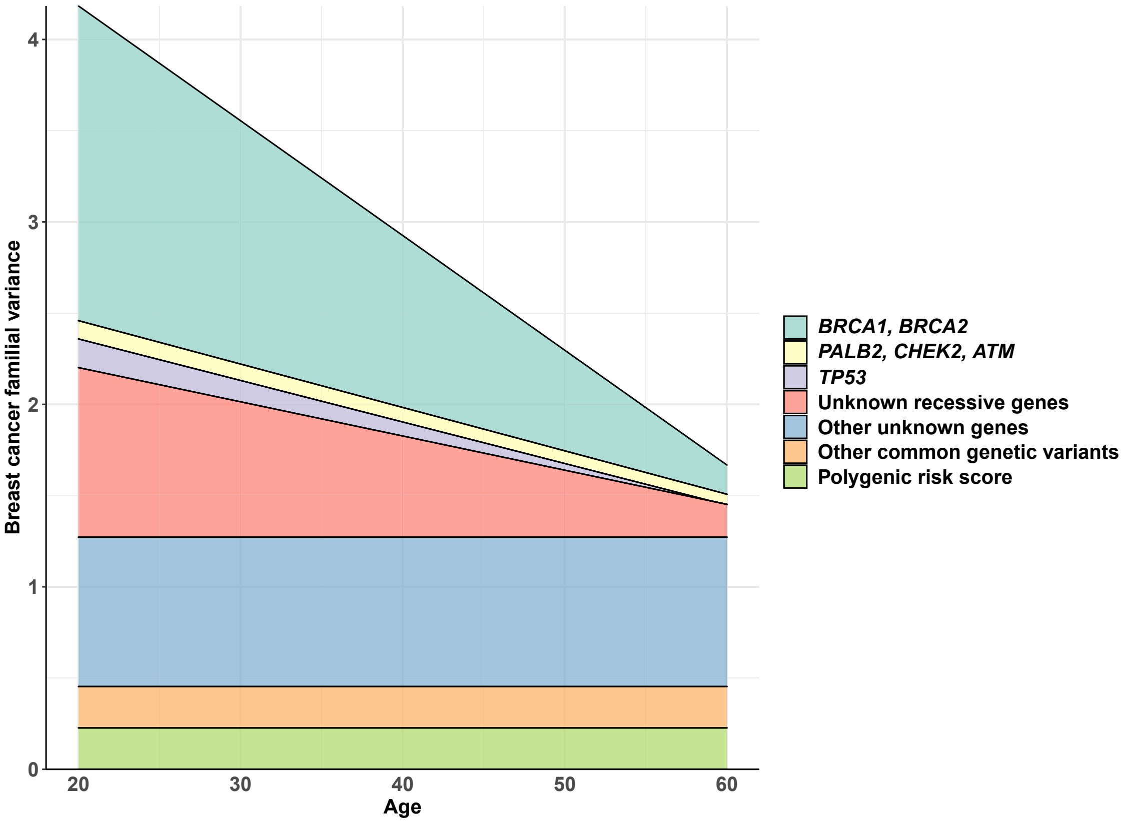

Figure 1. Age-specific breast cancer familial variance explained by genes
For a gene, the age-specific breast cancer familial variance explained by the gene was calculated as the age-specific difference in the $\sigma_P^2(t)$ between the model without the gene and the model with the gene. The variance explained by *BRCA1* and *BRCA2* was the $\sigma_P^2(t)$ of the model including an age-decreasing $\sigma_P^2(t)$ only minus the $\sigma_P^2(t)$ of the model including *BRCA1*, *BRCA2*, and an age-decreasing $\sigma_P^2(t)$. The variance explained by *PALB2*, *CHEK2*, and *ATM* was the $\sigma_P^2(t)$ of the model including *BRCA1*, *BRCA2*, and an age-decreasing $\sigma_P^2(t)$ minus the $\sigma_P^2(t)$ of the model including *BRCA1*, *BRCA2*, *PALB2*, *CHEK2*, *ATM*, and an age-decreasing $\sigma_P^2(t)$. The variance explained by *TP53* was the $\sigma_P^2(t)$ of the model including *BRCA1*, *BRCA2*, *PALB2*, *CHEK2*, *ATM*, and an age-decreasing $\sigma_P^2(t)$ minus the $\sigma_P^2(t)$ of the model including *BRCA1*, *BRCA2*, *PALB2*, *CHEK2*, *ATM*, *TP53*, and an age-decreasing $\sigma_P^2(t)$. The variance explained by unknown genes (recessive inheritance model) was the $\sigma_P^2(t)$ of the model including *BRCA1*, *BRCA2*, *PALB2*, *CHEK2*, *ATM*, *TP53*, and an age-decreasing $\sigma_P^2(t)$ minus the $\sigma_P^2(t)$ of the model including *BRCA1*, *BRCA2*, *PALB2*, *CHEK2*, *ATM*, *TP53*, the hypothetical gene, and an age-constant $\sigma_P^2(t)$. The variance explained by the polygenic risk score was from Mavaddat et al.[11] The variance explained by other common genetic variants was that explained by all imputable common genetic variants via the OncoArray (i.e., chip heritability), which was approximately twice that explained by known common genetic variants,[31] minus those explained by known common genetic variants, i.e., the polygenic risk score.

Most of the difference in log-likelihoods between the dominant inheritance model for the hypothetical gene and its equivalent nested model without the gene was attributed to a small number of families (Figure S1). For all ten families contributing the most evidence for the hypothetical gene, the proband was diagnosed with breast cancer before age 42 years, and all the affected relatives were diagnosed before age 37 years (Table S7). For three of these families, the proband carried a PV in *TP53*. No PVs in the other genes were identified for the probands of these families from the gene-panel testing data. We therefore hypothesized that the hypothetical gene might reflect the effects of *TP53* PVs and further extended the models to include *TP53*.

Incorporating *TP53* in addition to the five major genes while fitting an age-decreasing $\sigma_P^2(t)$ improved the model fit ($p < 10^{-15}$; Table S8). The best fitting model included a *TP53* RR, which decreased linearly with age on the log-RR scale over age 20–49 years and then was constant over age 50–79 years. Under this model, the frequency for *TP53* PVs was 0.017% (95% CI: 0.009%, 0.034%) and the estimated cumulative risk of breast cancer to age 80 years for *TP53* PVs was 45.0% (95% CI: 25.5%, 74.0%) (Figure 2). After fitting *TP53*, there was marginal evidence ($p = 0.07$) that $\sigma_P^2(t)$ decreased with age. On the basis of the model with an age-decreasing $\sigma_P^2(t)$, the proportion of the total breast cancer familial variance attributed to *TP53* was 3.5% at age 20–29 years and decreased with age thereafter; in terms of the residual familial variance after adjusting for the effects of *BRCA1*, *BRCA2*, *PALB2*, *CHEK2*, and *ATM*, the proportion decreased from 6.1% at age 20–29 years (Figure 1; Table S6).

On the basis of the best fitting model that included *TP53* and an age-constant $\sigma_P^2(t)$, we incorporated an additional hypothetical gene (Table 2). There was evidence for the hypothetical gene under both the recessive and general

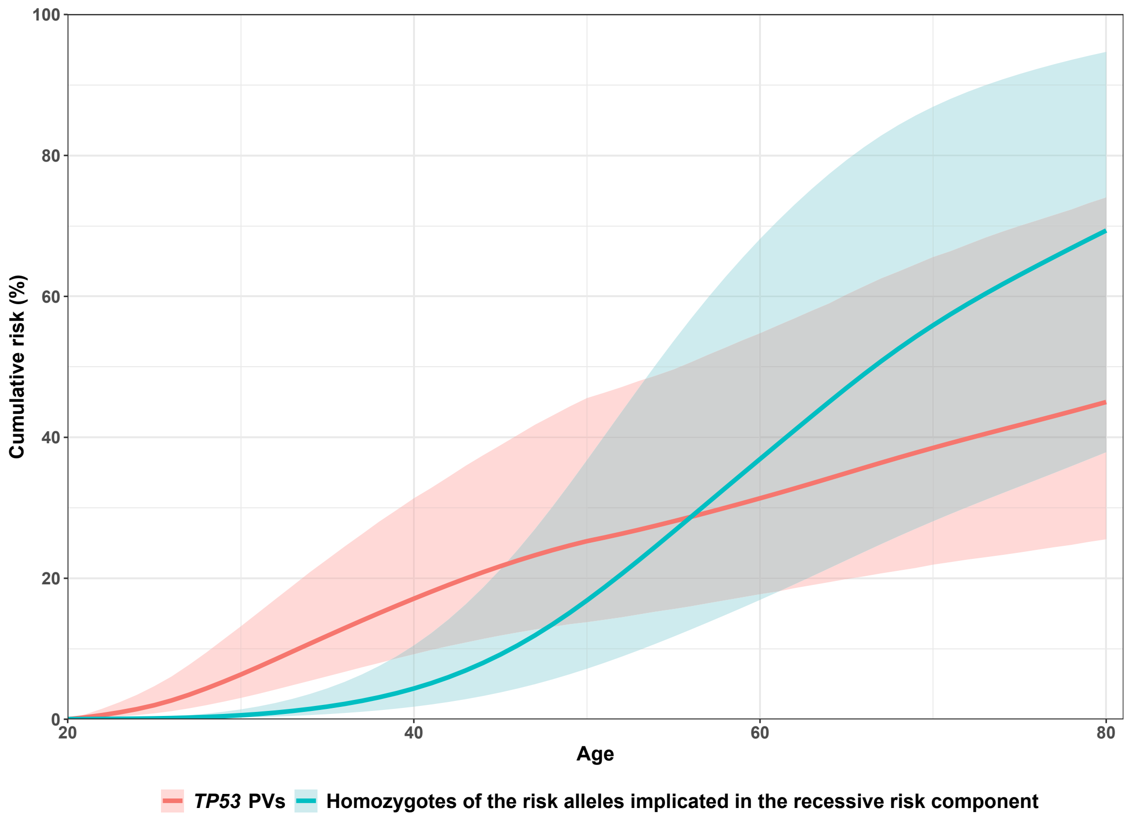

Figure 2. Age-specific cumulative risks with 95% confidence intervals for *TP53* PVs and homozygotes of the risk alleles implicated in the recessive risk component
The recessive risk component is from the best fit model including *BRCA1*, *BRCA2*, *PALB2*, *CHEK2*, *ATM*, *TP53*, a seventh hypothetical gene, and an age-constant $\sigma_P^2(t)$. Shaded areas are 95% confidence intervals.

inheritance models (both $p < 0.02$), and the general inheritance model essentially converged to the recessive inheritance model. Under the best fitting recessive inheritance model, the hypothetical gene had a risk allele frequency of 13% (95% CI: 5%, 23%) and an RR of 10 (95% CI: 4, 25), giving a cumulative risk to age 80 of 69.4% (95% CI: 37.9%, 94.7%) for homozygotes (Figure 2). Under this model, $\sigma_P^2(t)$ was estimated to be 1.27 (95% CI: 0.94, 1.65) and there was no evidence that it depended on age ($p = 0.81$). The proportion of the total breast cancer familial variance explained by the hypothetical gene was 21.6% at age 20–29 years and decreased steadily with age thereafter; in terms of the residual familial variance after adjusting for the effects of *BRCA1*, *BRCA2*, *PALB2*, *CHEK2*, *ATM*, and *TP53*, the proportion was 39.7% at age 20–29 years and decreased thereafter (Figure 1; Table S6). Under this model, the PV allele frequencies of *BRCA1*, *BRCA2*, *PALB2*, *CHEK2*, *ATM*, and *TP53* were 0.080%, 0.141%, 0.059%, 0.385%, 0.167%, and 0.017%, respectively, corresponding to frequencies of individuals with *BRCA1*, *BRCA2*, *PALB2*, *CHEK2*, *ATM*, and *TP53* PVs of 0.159%, 0.281%, 0.119%, 0.768%, 0.334%, and 0.034%, respectively.

No family contributed a large change in the log-likelihood when the hypothetical gene (recessive inheritance model) was added (Figure S1). For all ten families contributing most to the evidence for the hypothetical gene, the proband had at least one sister diagnosed with breast cancer before or at age 45 years (Table S9). No PV was identified for the probands of these ten families from the gene-panel testing data.

Similar results were found from the sensitivity analyses in which the PV test sensitivity was reduced to 80% (Table S10). With this sensitivity, the PV allele frequencies of *BRCA1*, *BRCA2*, *PALB2*, *CHEK2*, *ATM*, and *TP53* genes were 0.089%, 0.157%, 0.067%, 0.432%, 0.187%, and 0.020%, respectively, corresponding to frequencies of individuals with *BRCA1*, *BRCA2*, *PALB2*, *CHEK2*, *ATM*, and *TP53* PVs of 0.178%, 0.313%, 0.133%, 0.862%, 0.374%, and 0.039%, respectively.

The age-specific FRRs associated with a family history in a first-degree relative predicted by the best fitting recessive inheritance model were consistent with those observed by the largest combined analysis of epidemiological studies[1] (Table 3). At each age, the FRR associated with a sister affected was slightly greater than the FRR associated with the mother affected.

Discussion

This study provides insights into the genetic susceptibility of breast cancer. In terms of explaining why women of the

Table 2. Models including *BRCA1*, *BRCA2*, *PALB2*, *CHEK2*, *ATM*, *TP53*, a hypothetical gene, and a polygenic component

Parameters	Dominant inheritance model	Recessive inheritance model[a]	General inheritance model	Recessive inheritance model with an age-dependent $\sigma_P^2(t)$[b]
$\sigma_P^2(t)$ (95% CI)	1.534 (1.448, 1.624)	1.272 (0.944, 1.649)	1.272 (0.908, 1.698)	$\alpha = 1.066$ (95% CI: −1.186, 3.318), $\beta = 0.004$ (95% CI: −0.033, 0.040)
**PV allele frequency (95% CI)**				
BRCA1	0.080% (0.069%, 0.092%)	0.080% (0.069%, 0.092%)	0.080% (0.069%, 0.092%)	0.080% (0.069%, 0.092%)
BRCA2	0.141% (0.126%, 0.158%)	0.141% (0.125%, 0.158%)	0.141% (0.125%, 0.158%)	0.141% (0.125%, 0.158%)
PALB2	0.060% (0.049%, 0.073%)	0.059% (0.049%, 0.073%)	0.059% (0.049%, 0.073%)	0.059% (0.049%, 0.073%)
CHEK2	0.385% (0.338%, 0.438%)	0.385% (0.338%, 0.438%)	0.385% (0.338%, 0.438%)	0.385% (0.338%, 0.438%)
ATM	0.167% (0.139%, 0.200%)	0.167% (0.139%, 0.200%)	0.167% (0.139%, 0.200%)	0.167% (0.139%, 0.200%)
TP53	0.018% (0.005%, 0.057%)	0.017% (0.008%, 0.038%)	0.017% (0.008%, 0.037%)	0.017% (0.008%, 0.038%)
Hypothetical gene	0.001% (0.0002%, 0.007%)	13.1% (5.4%, 23.2%)	13.1% (4.6%, 26.2%)	13.1% (5.4%, 23.2%)
**RR of *TP53* PVs (95% CI)**				
Age 20–29 years	134.43 (44.84, 401.30)	142.86 (62.37, 325.81)	142.86 (63.10, 321.86)	144.90 (56.64, 368.97)
Age 30–39 years	30.81 (9.59, 98.44)	32.10 (14.81, 70.02)	32.10 (15.01, 69.05)	32.45 (13.21, 79.73)
Age 40–49 years	7.06 (1.58, 31.60)	7.21 (2.42, 21.48)	7.21 (2.49, 21.10)	7.27 (2.25, 23.50)
Age 50–59 years	3.01 (0.36, 25.45)	3.13 (0.87, 11.29)	3.13 (0.90, 10.92)	3.16 (0.68, 14.75)
Age 60–69 years	3.01 (0.36, 25.45)	3.13 (0.87, 11.29)	3.13 (0.90, 10.92)	3.16 (0.68, 14.75)
Age 70–79 years	3.01 (0.36, 25.45)	3.13 (0.87, 11.29)	3.13 (0.90, 10.92)	3.16 (0.68, 14.75)
**RR of the hypothetical gene (95% CI)**				
Heterozygote	282.55 (259.43, 307.72)	1	1	1
Homozygote	282.55 (259.43, 307.72)	10.03 (4.04, 24.9)	10.03 (3.47, 28.97)	10.42 (4.62, 23.53)
Log likelihood	−35,635.90	−35,632.18	−35,632.09	−35,632.06
Number of parameters estimated	12	12	13	13
Akaike information criterion	71,295.80	71,288.18	71,290.18	71,290.12
p[c]	0.23	0.005	0.01	0.01
Best fitting model	no	yes	no	no

CI: confidence interval; PV: pathogenic variant; RR: relative risk.
[a]Log-RR = $\alpha + \beta \times (\text{age} - 20)$ in age 20–49 years, where $\alpha = 5.71$ (95% CI: 4.72, 6.69), $\beta = -0.15$ (95% CI: −0.21, −0.09).
[b]$\sigma_P^2(t) = \alpha + \beta \times \text{age}$.
[c]From the likelihood ratio test of comparing with the model including *BRCA1*, *BRCA2*, *PALB2*, *CHEK2*, *ATM*, *TP53*, and an age-constant $\sigma_P^2(t)$.

same age differ in risk, the known susceptibility genes, including *BRCA1*, *BRCA2*, *PALB2*, *CHEK2*, *ATM*, and *TP53* play more important roles at younger ages; the proportion of the total breast cancer familial variance explained by these genes was 46% at age 20–29 years and decreased steadily with age thereafter. GWASs have identified several hundred common genetic variants associated with breast cancer risk;[31] these variants combine multiplicatively, and their effects can thus be summarized by a PRS. The most extensively validated PRS, comprising 313 common genetic variants, is associated with breast cancer risk with little evidence of variation in the relative risk by age,[11] implying that the PRS explains a higher fraction of the breast cancer familial variance at older ages than at younger ages.[32,33] The odds ratio (OR) per standard deviation of the PRS is 1.61, equivalent to a familial variance of ~0.23. Therefore, the PRS accounts for about 20% of the residual polygenic variance of 1.27 estimated after taking into account the known and predicted additional major gene(s), in line with previous estimates for the PRS contribution.[11,20] The breast cancer familial variance explained by all imputable common genetic variants via the OncoArray (i.e., chip heritability) was estimated to be approximately twice that explained by the known common genetic variants (and thus about 40% of the residual polygenic variance).[31] As illustrated in Figure 1, there is substantial breast cancer familial variance that cannot be explained by the known susceptibility genes and common genetic variants, and the unexplained familial variance is larger at younger ages. Therefore, breast cancer genomic studies focused on individuals diagnosed at younger ages may be a fruitful approach to identifying novel breast

Table 3. Age-specific breast cancer familial relative risks associated with an affected first-degree relative

Age (years)	Predicted familial relative risk with the mother affected	Predicted familial relative risk with a sister affected	Observed familial relative risk (95% CI)[a]
25	9.90	10.35	5.7 (2.7, 11.8)
30	5.84	6.22	
35	3.37	3.71	
40	2.44	2.74	2.0 (1.5, 2.8)
45	2.07	2.31	
50	1.82	2.00	1.6 (1.2, 2.1)
55	1.67	1.80	
60	1.56	1.65	1.4 (1.2, 1.7)
65	1.49	1.55	
70	1.44	1.48	
75	1.39	1.42	

CI, confidence interval.
[a]From the epidemiological studies by Collaborative Group on Hormonal Factors in Breast Cancer.[1]

cancer genetic susceptibility genes or variants. Our analyses predicted a recessively inherited risk component explaining a substantial proportion of the residual familial variance after taking into account PVs in *BRCA1*, *BRCA2*, *PALB2*, *CHEK2*, *ATM*, *TP53*, and the PRS, with the proportion greater at younger ages (44% at ages 20–29 years) and decreased steadily with age thereafter.

Although our study sample overlapped with the data used in three previous analyses,[12–14] the current dataset was seven times larger. Moreover, we were able to include PV data for the large majority of probands, providing much more robust analyses. Our evidence for an additional recessively inherited major breast cancer-susceptibility gene, after considering the effects of PVs in *BRCA1*, *BRCA2*, *PALB2*, *CHEK2*, *ATM*, and *TP53*, is consistent with the results from previous analyses.[12–15] The best fitting model predicted age-specific FRRs consistent with the observed FRRs by epidemiological studies. The recessive risk component leads to the prediction that the FRR will be higher for sisters of affected women than for mothers of affected daughters, and this is consistent with observations in population-based epidemiological studies.[1,13,25,34–36] Although the lower relative risk for mothers could be partly explained by them being parous by definition, the risk difference is more profound before age 50 years when mothers are pre-menopausal and the protective effect of parity is weaker.[1,35] Our analysis used birth-cohort-specific incidences; therefore, the results are unlikely to be due to the higher incidence for sisters, who were born more recently than mothers. However, we cannot rule out the possibility that part of the increased risk to sisters may be due to surveillance because sisters of individuals with breast cancer are more likely to participate in screening programs.

We also cannot rule out the role of non-genetic familial factors in explaining familial aggregation of breast cancer, as we only considered genetic models. The Nordic Twin Study of breast cancer found evidence for non-genetic effects shared by twins, especially at younger ages, which would also be consistent with that the FRR is greater for sister pairs than for mother-daughter pairs[30] and could reflect factors operating prior to adulthood such as puberty-related risk factors.

Our results also do not necessarily imply there is a single additional susceptibility gene (indeed this is highly unlikely), nor imply one or two genes contribute most of the effect, because such gene(s) would probably have been identified through linkage or association studies. It is more likely that the recessive component reflects the combined effects of multiple variants (and in more than one gene or non-coding region), each potentially being associated with different effects, although the actual number of variants and genes cannot be inferred from segregation analysis alone. Nevertheless, our results could inform the design of sequencing studies to try to identify such variants by focusing on analyzing the following types of samples that do not have PVs in the known genes: (1) families with multiple affected siblings; (2) families of young women (e.g., ≤ 40 years) with breast cancer; and (3) a combination of (1) and (2).

Breast cancer FRR depends on pathological subtype, and the risks associated with both known breast cancer-susceptibility genes and PRS differ across subtypes.[11,34,37] However, our study was not able to investigate this in detail because, for most families, tumor features were only available for the affected probands. Further analyses of datasets with breast cancer subtype data for both probands and relatives are needed to investigate the extent to which the recessive risk component and the residual polygenic variance are subtype specific.

We calculated the age-specific proportion of breast cancer familial variance explained by PVs in major genes as the difference in the polygenic variance between nested

models. The results in Table S6 suggest the proportion at age 60 and older might be minimal, despite the fact the RRs for breast cancer associated with PVs at those ages are greater than 1. The estimated contribution of PVs at older ages may have been underestimated due to the fact that the total breast cancer familial variance used to calculate the contributions was based on a model without any major genes. Under this model, the polygenic variance decreased linearly with age, which may have resulted in imprecision of the estimated variance at older ages.

Although our study—by design—did not include probands diagnosed after age 70 years, the analyses are still informative for familial aggregation after age 70 years because it was the phenotypes (i.e., age of cancer diagnosis or censoring) of relatives, not of probands, that contributed to the analyses, as the likelihood of observing the family phenotypes was conditioned on the proband's phenotype. Our study included 22,253 relatives from 12,393 families (71% of the total families) who were censored after age 70 years, and 470 of them were diagnosed with breast cancer.

Our study did not consider the PVs in other recently established breast cancer-susceptibility genes such as *BARD1*, *RAD51C*, and *RAD51D*, which are incorporated in the most recent version of BOADICEA (version 6).[21] However, these PVs only explain on average 0.31% of residual breast cancer familial variance,[21] so not considering these PVs is unlikely to have materially impacted our results.

We previously conducted a population-based study of women with breast cancer diagnosed at a young age in ABCFR and confirmed that germline *TP53* PVs occur among women diagnosed at a very young age.[38] Here, we estimated the PV frequency to be 0.017%, similar to the 1/5,000 estimated by a segregation analysis of 278 breast cancer families ascertained via population-based cases diagnosed before age 30 years.[39] Reliable breast cancer risk estimates from population-based studies associated with *TP53* PVs are lacking. Two studies of sarcoma families reported a breast cancer cumulative risk for *TP53* PVs of approximately 37% to age 80 years and 54% to age 70 years, respectively.[40,41] Although these studies did not report confidence intervals for their risk estimates, our confidence intervals include those point risk estimates and therefore are not significantly different. Another prospective analysis from the longitudinal Li-Fraumeni Syndrome Study reported a breast cancer cumulative risk of approximately 56% to age 60 years, higher than our estimate and outside our confidence intervals.[42] However, the participants in that study were from families with Li-Fraumeni syndrome, who were ascertained on the basis of family history of cancer, including family history of breast cancer. That was a prospective study with no adjustments for the ascertainment of study participants. Therefore, the risk estimate of the longitudinal Li-Fraumeni Syndrome Study would be applicable to women with *TP53* PVs with cancer family history. On the other hand, the estimates in our study are based on families from population-based studies of breast cancer, and our analysis has adjusted for the ascertainment process and allowed for the modification of cancer risks by the residual familial component. As a result, our estimate represents the average risk for women with *TP53* PVs unselected for family history and would be expected to be lower than the estimates for those with family history of breast cancer—similar to the patterns observed previously for the risks for women with *BRCA1/2* PVs.[3,43] Since the *TP53* PV risks are assumed to be modified by the residual familial component, the breast cancer risk predicted by the model will vary by the exact family history, and the risks for those with one or two affected relatives will be higher than the average risk.

The findings of the current study can be used to update breast cancer-risk models, in particular BOADICEA, which currently considers the effects of PVs in known major genes and a polygenic component.[21] Thus, the model could be updated by incorporating the recessive component, using the revised estimate for the polygenic variance, and adding *TP53* PVs. However, the extent to which including the additional genes and using the updated polygenic variance improves breast cancer-risk prediction overall needs to be investigated. Moreover, such a revised model would need to be validated with independent studies.

To allow for the possibility that not all PVs can be detected by the test methods used, our analysis included a test sensitivity parameter, which was assumed to be ~90%. The actual sensitivity is difficult to estimate and will depend on the methods used. The current implementation of BOADICEA assumes sensitivities of 89%, 96%, 92%, 98%, and 94% for *BRCA1*, *BRCA2*, *PALB2*, *CHEK2*, and *ATM*, respectively, on the basis of typical clinically testing approaches,[2,21] but the sensitivity will be lower for research testing, particularly because large rearrangements would not have been detected in the targeted sequencing used in SEARCH.[2] Using lower test sensitivities might have resulted in some underestimation of the polygenic component and some overestimation of the contributions of rare PVs to familial aggregation, through the estimation of higher PV frequencies. However, the differences would be small, as our estimates of PV frequencies for *BRCA1* and *BRCA2* were slightly greater than, and the estimates for *PALB2*, *CHEK2*, and *ATM* were similar to, those assumed in the BOADICEA, which had been derived from large population-based targeted-sequencing data and adjusted for the test sensitivity of targeted-sequencing.[21] On the other hand, by using lower test sensitivities, our analysis reduced the possibility that the recessive risk component is simply due to unidentified variants in any of the considered major genes; our more conservative sensitivity analysis assuming the test sensitivity to be 80% still provided evidence for a recessive risk component.

Our study has several strengths. First, it included data from more than 17,000 three-generational families ascertained via probands from population-based studies and screened for high- and moderate-risk PVs in the major

known susceptibility genes. Second, our study incorporated the explicit age-specific effects of PVs in *PALB2*, *CHEK2*, *ATM*, and *TP53* in addition to *BRCA1* and *BRCA2* while modeling the residual familial variance of breast cancer as a function of age with a polygenic component.

The study has also some limitations. First, family history in some families was self-reported and therefore subject to reporting errors, though reporting of breast cancer in first-degree relatives is generally considered to be accurate.[44] Second, we modeled the major genes by using a single locus with eight alleles and assumed the genes were in a dominant hierarchy rather than seven loci each with two alleles. While this approach substantially reduces the computational time, it could introduce some imprecision in the parameter estimates, although the impact is likely to be minimal because the PVs in the known genes are rare. The polygenic component was also approximated with a binomial distribution inherited under the hypergeometric model; previous analyses had found that results are insensitive to the number of loci assumed,[12] but this might not be true for more complex models. Third, the majority of our probands were of White European ancestry; therefore, our findings might not be applicable to other ethnicities.

In conclusion, by considering the explicit effects of established major breast cancer-susceptibility genes and polygenes and using the largest sample size of its kind, our analysis estimates the proportion of breast cancer familial aggregation that is explained by established susceptibility genes and variants and provides evidence for an additional recessive risk component, which could explain a substantial proportion of the residual familial aggregation, especially at a younger age. Our findings are informative for the design of sequencing studies to identify novel breast cancer-susceptibility genes and modeling breast cancer genetic susceptibility for disease risk prediction.

Data and code availability

The datasets supporting the current study have not been deposited in a public repository because of ethical requirements. Requests for access to the data analyzed in this study should be made to J.L.H. (ABCFR) and P.D.P. (SEARCH). Requests for access to the analytical code should be made to S.L. and A.C.A.

Supplemental information

Supplemental information can be found online at https://doi.org/10.1016/j.ajhg.2022.09.006.

Acknowledgments

We thank all the participants in this study, and the entire team, past and current investigators of ABCFR. The work was supported by Cancer Research UK grant PPRPGM-Nov20\100002, NIHR Cambridge Biomedical Research Centre, Victorian Cancer Agency grant ECRF19020, Picchi Award for Excellence in Cancer Research from the Victorian Comprehensive Cancer Centre, and the US National Institutes of Health (grant number RO1CA159868). S.L. is a Victorian Cancer Agency (VCA) early career research fellow (ECRF19020). T.N.-D. is a VCA mid-career research fellow (MCRF21029). M.C.S. is an NHMRC senior research fellow (APP1155163). J.L.H. is an NHMRC Senior Principal Research Fellow. Additional acknowledgements are in the supplemental information. This work was performed with resources provided by the Cambridge Service for Data Driven Discovery (CSD3) operated by the University of Cambridge Research Computing Service (www.csd3.cam.ac.uk), provided by Dell EMC and Intel with Tier-2 funding from the Engineering and Physical Sciences Research Council (capital grant EP/T022159/1) and DiRAC funding from the Science and Technology Facilities Council (www.dirac.ac.uk).

Declaration of interests

A.C.A., A.L., and D.F.E. are listed as creators of BOADICEA v.5, which has been licensed by Cambridge Enterprise. G.D.S. is an employee of Genetic Technologies Ltd.

Received: May 25, 2022
Accepted: September 12, 2022
Published: October 6, 2022

Web resources

OMIM, www.omim.org

References

1. Collaborative Group on Hormonal Factors in Breast Cancer (2001). Familial breast cancer: collaborative reanalysis of individual data from 52 epidemiological studies including 58, 209 women with breast cancer and 101, 986 women without the disease. Lancet *358*, 1389–1399.
2. Breast Cancer Association Consortium, Carvalho, S., González-Neira, A., Wahlström, C., Parsons, M.T., Wang, Q., Dennis, J., Alonso, M.R., Herraez, B., Núñez-Torres, R., et al. (2021). Breast Cancer Risk Genes - Association Analysis in More than 113, 000 Women. N. Engl. J. Med. *384*, 428–439.
3. Kuchenbaecker, K.B., Hopper, J.L., Barnes, D.R., Phillips, K.A., Mooij, T.M., Roos-Blom, M.J., Jervis, S., van Leeuwen, F.E., Milne, R.L., Andrieu, N., et al. (2017). Risks of breast, ovarian, and contralateral breast cancer for BRCA1 and BRCA2 mutation carriers. JAMA *317*, 2402–2416.
4. Antoniou, A.C., Casadei, S., Heikkinen, T., Barrowdale, D., Pylkäs, K., Roberts, J., Lee, A., Subramanian, D., De Leeneer, K., Fostira, F., et al. (2014). Breast-cancer risk in families with mutations in PALB2. N. Engl. J. Med. *371*, 497–506.
5. Yang, X., Leslie, G., Doroszuk, A., Schneider, S., Allen, J., Decker, B., Dunning, A.M., Redman, J., Scarth, J., Plaskocinska, I., et al. (2020). Cancer Risks Associated With Germline PALB2 Pathogenic Variants: An International Study of 524 Families. J. Clin. Oncol. *38*, 674–685.
6. Weischer, M., Bojesen, S.E., Ellervik, C., Tybjaerg-Hansen, A., and Nordestgaard, B.G. (2008). CHEK2*1100delC genotyping for clinical assessment of breast cancer risk: meta-analyses of 26, 000 patient cases and 27, 000 controls. J. Clin. Oncol. *26*, 542–548.

7. Schmidt, M.K., Hogervorst, F., van Hien, R., Cornelissen, S., Broeks, A., Adank, M.A., Meijers, H., Waisfisz, Q., Hollestelle, A., Schutte, M., et al. (2016). Age- and Tumor Subtype-Specific Breast Cancer Risk Estimates for CHEK2*1100delC Carriers. J. Clin. Oncol. *34*, 2750–2760.
8. Thompson, D., Duedal, S., Kirner, J., McGuffog, L., Last, J., Reiman, A., Byrd, P., Taylor, M., and Easton, D.F. (2005). Cancer risks and mortality in heterozygous ATM mutation carriers. J. Natl. Cancer Inst. *97*, 813–822.
9. Easton, D.F., Pharoah, P.D.P., Antoniou, A.C., Tischkowitz, M., Tavtigian, S.V., Nathanson, K.L., Devilee, P., Meindl, A., Couch, F.J., Southey, M., et al. (2015). Gene-panel sequencing and the prediction of breast-cancer risk. N. Engl. J. Med. *372*, 2243–2257.
10. Hu, C., Hart, S.N., Gnanaolivu, R., Huang, H., Lee, K.Y., Na, J., Gao, C., Lilyquist, J., Yadav, S., Boddicker, N.J., et al. (2021). A Population-Based Study of Genes Previously Implicated in Breast Cancer. N. Engl. J. Med. *384*, 440–451.
11. Mavaddat, N., Michailidou, K., Dennis, J., Lush, M., Fachal, L., Lee, A., Tyrer, J.P., Chen, T.H., Wang, Q., Bolla, M.K., et al. (2019). Polygenic Risk Scores for Prediction of Breast Cancer and Breast Cancer Subtypes. Am. J. Hum. Genet. *104*, 21–34.
12. Antoniou, A.C., Pharoah, P.D., McMullan, G., Day, N.E., Ponder, B.A., and Easton, D. (2001). Evidence for further breast cancer susceptibility genes in addition to BRCA1 and BRCA2 in a population-based study. Genet. Epidemiol. *21*, 1–18.
13. Cui, J., Antoniou, A.C., Dite, G.S., Southey, M.C., Venter, D.J., Easton, D.F., Giles, G.G., McCredie, M.R., and Hopper, J.L. (2001). After BRCA1 and BRCA2-what next? Multifactorial segregation analyses of three-generation, population-based Australian families affected by female breast cancer. Am. J. Hum. Genet. *68*, 420–431.
14. Antoniou, A.C., Pharoah, P.D.P., McMullan, G., Day, N.E., Stratton, M.R., Peto, J., Ponder, B.J., and Easton, D.F. (2002). A comprehensive model for familial breast cancer incorporating BRCA1, BRCA2 and other genes. Br. J. Cancer *86*, 76–83.
15. Kaufman, D.J., Beaty, T.H., and Struewing, J.P. (2003). Segregation Analysis of 231 Ashkenazi Jewish Families for Evidence of Additional Breast Cancer Susceptibility Genes. Cancer Epidemiol. Biomarkers Prev. *12*, 1045–1052.
16. Antoniou, A.C., Pharoah, P.P.D., Smith, P., and Easton, D.F. (2004). The BOADICEA model of genetic susceptibility to breast and ovarian cancer. Br. J. Cancer *91*, 1580–1590.
17. Antoniou, A.C., Cunningham, A.P., Peto, J., Evans, D.G., Lalloo, F., Narod, S.A., Risch, H.A., Eyfjord, J.E., Hopper, J.L., Southey, M.C., et al. (2008). The BOADICEA model of genetic susceptibility to breast and ovarian cancers: updates and extensions. Br. J. Cancer *98*, 1457–1466.
18. Lee, A.J., Cunningham, A.P., Kuchenbaecker, K.B., Mavaddat, N., Easton, D.F., Antoniou, A.C.; Consortium of Investigators of Modifiers of BRCA1/2; and Breast Cancer Association Consortium (2014). BOADICEA breast cancer risk prediction model: updates to cancer incidences, tumour pathology and web interface. Br. J. Cancer *110*, 535–545.
19. Lee, A.J., Cunningham, A.P., Tischkowitz, M., Simard, J., Pharoah, P.D., Easton, D.F., and Antoniou, A.C. (2016). Incorporating truncating variants in PALB2, CHEK2, and ATM into the BOADICEA breast cancer risk model. Genet. Med. *18*, 1190–1198.
20. Lee, A., Mavaddat, N., Wilcox, A.N., Cunningham, A.P., Carver, T., Hartley, S., Babb de Villiers, C., Izquierdo, A., Simard, J., Schmidt, M.K., et al. (2019). BOADICEA: a comprehensive breast cancer risk prediction model incorporating genetic and nongenetic risk factors. Genet. Med. *21*, 1708–1718.
21. Lee, A., Mavaddat, N., Cunningham, A.P., Carver, T., Archer, S., Walter, F.M., Tischkowitz, M., Roberts, J., Usher-Smith, J., Simard, J., et al. (2022). Enhancing the BOADICEA cancer risk prediction model to incorporate new data on RAD51C, RAD51D, BARD1, updates to tumour pathology and cancer incidences. Preprint at medRxiv. https://doi.org/10.1101/2022.01.27.22269825.
22. Hopper, J.L., Giles, G.G., McCredie, M.R.E., and Boyle, P. (1994). Background, rationale and protocol for a case-control-family study of breast cancer. Breast *3*, 79–86.
23. McCredie, M.R., Dite, G.S., Giles, G.G., and Hopper, J.L. (1998). Breast cancer in Australian women under the age of 40. Cancer Causes Control. *9*, 189–198.
24. Hopper, J.L., Chenevix-Trench, G., Jolley, D.J., Dite, G.S., Jenkins, M.A., Venter, D.J., McCredie, M.R.E., and Giles, G.G. (1999). Design and analysis issues in a population-based, case-control-family study of the genetic epidemiology of breast cancer and the Co-operative Family Registry for Breast Cancer Studies (CFRBCS). JNCI Monographs, 95–100.
25. Pharoah, P.D., Lipscombe, J.M., Redman, K.L., Day, N.E., Easton, D.F., and Ponder, B.A. (2000). Familial predisposition to breast cancer in a British population: implications for prevention. Eur. J. Cancer *36*, 773–779.
26. Southey, M.C., Dowty, J.G., Riaz, M., Steen, J.A., Renault, A.L., Tucker, K., Kirk, J., James, P., Winship, I., Pachter, N., et al. (2021). Population-based estimates of breast cancer risk for carriers of pathogenic variants identified by gene-panel testing. NPJ Breast Cancer *7*, 153.
27. Lange, K. (1997). An approximate model of polygenic inheritance. Genetics *147*, 1423–1430.
28. Fernando, R.L., Stricker, C., and Elston, R.C. (1994). The finite polygenic mixed model: An alternative formulation for the mixed model of inheritance. Theor. Appl. Genet. *88*, 573–580.
29. Pharoah, P.D.P., Antoniou, A., Bobrow, M., Zimmern, R.L., Easton, D.F., and Ponder, B.A.J. (2002). Polygenic susceptibility to breast cancer and implications for prevention. Nat. Genet. *31*, 33–36.
30. Möller, S., Mucci, L.A., Harris, J.R., Scheike, T., Holst, K., Halekoh, U., Adami, H.O., Czene, K., Christensen, K., Holm, N.V., et al. (2016). The Heritability of Breast Cancer among Women in the Nordic Twin Study of Cancer. Cancer Epidemiol. Biomarkers Prev. *25*, 145–150.
31. Michailidou, K., Lindström, S., Dennis, J., Beesley, J., Hui, S., Kar, S., Lemaçon, A., Soucy, P., Glubb, D., Rostamianfar, A., et al. (2017). Association analysis identifies 65 new breast cancer risk loci. Nature *551*, 92–94.
32. Li, S. (2021). Negative Age-Dependence of the Polygenic Risk Score Gradient for Colorectal Cancer. Gastroenterology *160*, 2214–2215.
33. Li, S., and Hopper, J.L. (2021). Age dependency of the polygenic risk score for colorectal cancer. Am. J. Hum. Genet. *108*, 525–526.
34. Mavaddat, N., Pharoah, P.D., Blows, F., Driver, K.E., Provenzano, E., Thompson, D., Macinnis, R.J., Shah, M., SEARCH Team, and Antoniou, A.C. (2010). Familial relative risks for breast cancer by pathological subtype: a population-based cohort study. Breast Cancer Res. *12*, R10.
35. Dite, G.S., Whittemore, A.S., Knight, J.A., John, E.M., Milne, R.L., Andrulis, I.L., Southey, M.C., McCredie, M.R.E., Giles,

G.G., Miron, A., et al. (2010). Increased cancer risks for relatives of very early-onset breast cancer cases with and without BRCA1 and BRCA2 mutations. Br. J. Cancer *103*, 1103–1108.
36. Peto, J., Easton, D.F., Matthews, F.E., Ford, D., and Swerdlow, A.J. (1996). Cancer mortality in relatives of women with breast cancer: the OPCS Study. Office of Population Censuses and Surveys. Int. J. Cancer *65*, 275–283.
37. Breast Cancer Association Consortium, Dorling, L., Allen, J., Keeman, R., Dennis, J., Ahearn, T.U., Beckmann, M.W., Benitez, J., Blomqvist, C., Bojesen, S.E., et al. (2022). Pathology of Tumors Associated With Pathogenic Germline Variants in 9 Breast Cancer Susceptibility Genes. JAMA Oncol. *8*, e216744.
38. Mouchawar, J., Korch, C., Byers, T., Pitts, T.M., Li, E., McCredie, M.R.E., Giles, G.G., Hopper, J.L., and Southey, M.C. (2010). Population-based estimate of the contribution of TP53 mutations to subgroups of early-onset breast cancer: Australian Breast Cancer Family Study. Cancer Res. *70*, 4795–4800.
39. Lalloo, F., Varley, J., Ellis, D., Moran, A., O'Dair, L., Pharoah, P., Evans, D.G.R.; and Early Onset Breast Cancer Study Group (2003). Prediction of pathogenic mutations in patients with early-onset breast cancer by family history. Lancet *361*, 1101–1102.
40. Shin, S.J., Dodd-Eaton, E.B., Peng, G., Bojadzieva, J., Chen, J., Amos, C.I., Frone, M.N., Khincha, P.P., Mai, P.L., Savage, S.A., et al. (2020). Penetrance of Different Cancer Types in Families with Li-Fraumeni Syndrome: A Validation Study Using Multicenter Cohorts. Cancer Res. *80*, 354–360.
41. Mai, P.L., Best, A.F., Peters, J.A., DeCastro, R.M., Khincha, P.P., Loud, J.T., Bremer, R.C., Rosenberg, P.S., and Savage, S.A. (2016). Risks of first and subsequent cancers among TP53 mutation carriers in the National Cancer Institute Li-Fraumeni syndrome cohort. Cancer *122*, 3673–3681.
42. de Andrade, K.C., Khincha, P.P., Hatton, J.N., Frone, M.N., Wegman-Ostrosky, T., Mai, P.L., Best, A.F., and Savage, S.A. (2021). Cancer incidence, patterns, and genotype-phenotype associations in individuals with pathogenic or likely pathogenic germline TP53 variants: an observational cohort study. Lancet Oncol. *22*, 1787–1798.
43. Antoniou, A., Pharoah, P.D.P., Narod, S., Risch, H.A., Eyfjord, J.E., Hopper, J.L., Loman, N., Olsson, H., Johannsson, O., Borg, A., et al. (2003). Average risks of breast and ovarian cancer associated with BRCA1 or BRCA2 mutations detected in case Series unselected for family history: a combined analysis of 22 studies. Am. J. Hum. Genet. *72*, 1117–1130.
44. Murff, H.J., Spigel, D.R., and Syngal, S. (2004). Does this patient have a family history of cancer? An evidence-based analysis of the accuracy of family cancer history. JAMA *292*, 1480–1489.

The American Journal of Human Genetics, Volume *109*

Supplemental information

Segregation analysis of 17,425 population-based breast cancer families: Evidence for genetic susceptibility and risk prediction

Shuai Li, Robert J. MacInnis, Andrew Lee, Tu Nguyen-Dumont, Leila Dorling, Sara Carvalho, Gillian S. Dite, Mitul Shah, Craig Luccarini, Qin Wang, Roger L. Milne, Mark A. Jenkins, Graham G. Giles, Alison M. Dunning, Paul D.P. Pharoah, Melissa C. Southey, Douglas F. Easton, John L. Hopper, and Antonis C. Antoniou

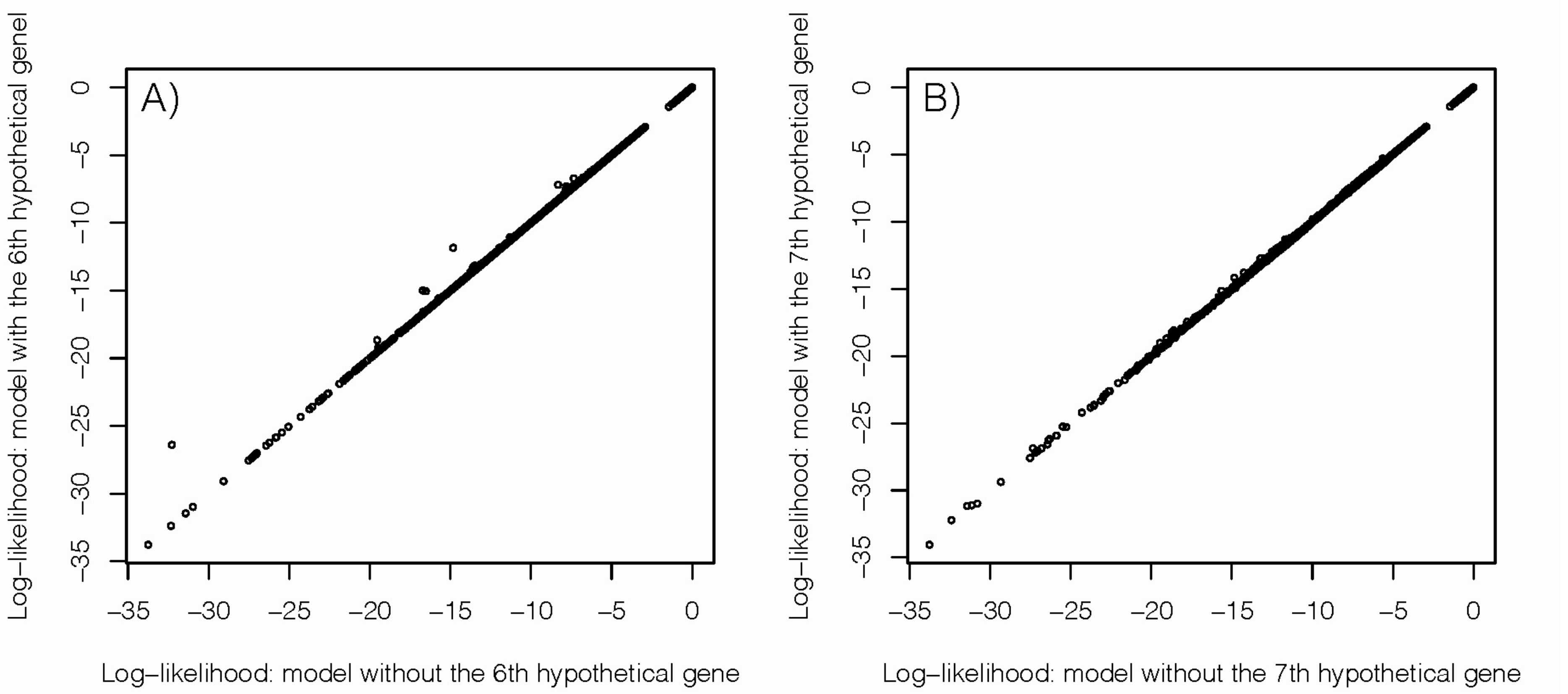

Figure S1 Log-likelihood changes for models in favour of the sixth and seventh hypothetical gene

A) Log-likelihood changes from the analysis of the sixth hypothetical gene. X axis is the log-likelihood of the model including *BRCA1*, *BRCA2*, *PALB2*, *CHEK2*, *ATM* and an age-constant $\sigma_P^2(t)$, and Y axis is the log-likelihood of the best fitting dominant inheritance model of the sixth hypothetical gene.

B) Log-likelihood changes from the analysis of the seventh hypothetical gene. X axis is the log-likelihood of the model including *BRCA1*, *BRCA2*, *PALB2*, *CHEK2*, *ATM*, *TP53* and an age-constant $\sigma_P^2(t)$, and Y axis is the log-likelihood of the best fitting recessive inheritance model of the seventh hypothetical gene.

Table S1 Assumed sensitivity of the pathogenic variant test methods in the ABCFR

Method	Assumed sensitivity
Heteroduplex Analysis	70%
Protein Truncation Test (PTT)	90%
Predictive Sequencing	100%
2-Dimensional Gel Electrophoresis	70%
Automated DNA Sequencing	100%
Multiplex ligation-dependent probe amplification (MLPA)	100%
MYRIAD DNA Sequencing	100%
Manual DNA Sequencing	100%
Denaturing High Performance Liquid (DHPLC)	90%
PCR and Agarose Gel Electrophoresis	70%
5'Nuclease Assay (TaqMan)	95%
Gene-panel test	100%

The test sensitivity used in analysis was the sum of the sensitivities of the test methods used, weighted by the proportional lengths of the exons screened, multiplying by 90% if MLPA had not been conducted. If MLPA had been conducted, 10% multiplying the sensitivity of MLPA weighted by the proportional lengths of the exons screened was added.

Table S2 Age-specific breast and ovarian cancer RRs for BRCA1, BRCA2, PALB2 and CHEK2 PVs

Age	Breast cancer RRs				Ovarian cancer RRs	
	BRCA1	*BRCA2*	*PALB2*	*CHEK2*	*BRCA1*	*BRCA2*
0	1.0	1.0	1.0	1.0	1.0	1.0
1	1.0	1.0	1.0	1.0	1.0	1.0
2	1.0	1.0	1.0	1.0	1.0	1.0
3	1.0	1.0	1.0	1.0	1.0	1.0
4	1.0	1.0	1.0	1.0	1.0	1.0
5	1.0	1.0	1.0	1.0	1.0	1.0
6	1.0	1.0	1.0	1.0	1.0	1.0
7	1.0	1.0	1.0	1.0	1.0	1.0
8	1.0	1.0	1.0	1.0	1.0	1.0
9	1.0	1.0	1.0	1.0	1.0	1.0
10	1.0	1.0	1.0	1.0	1.0	1.0
11	1.0	1.0	1.0	1.0	1.0	1.0
12	1.0	1.0	1.0	1.0	1.0	1.0
13	1.0	1.0	1.0	1.0	1.0	1.0
14	1.0	1.0	1.0	1.0	1.0	1.0
15	1.0	1.0	1.0	1.0	1.0	1.0
16	1.0	1.0	1.0	1.0	1.0	1.0
17	1.0	1.0	1.0	1.0	1.0	1.0
18	1.0	1.0	1.0	1.0	1.0	1.0
19	1.0	1.0	1.0	1.0	1.0	1.0
20	33.0	20.9	14.6	3.7	1.0	1.0
21	33.8	20.7	14.3	3.6	1.0	1.0
22	34.6	20.5	14.0	3.6	1.0	1.0
23	35.5	20.3	13.7	3.5	1.0	1.0
24	36.4	20.2	13.4	3.5	1.0	1.0
25	37.2	20.0	13.1	3.4	1.0	1.0
26	38.2	19.8	12.9	3.4	1.0	1.0
27	39.1	19.6	12.6	3.3	1.0	1.0
28	40.0	19.5	12.3	3.3	1.0	1.0
29	41.0	19.3	12.1	3.2	1.0	1.0
30	42.0	19.1	11.8	3.2	11.1	1.0
31	38.9	18.3	11.6	3.1	13.6	1.0
32	36.0	17.5	11.3	3.1	16.5	1.0
33	33.3	16.7	11.1	3.1	20.2	1.0
34	30.8	16.0	10.9	3.0	24.6	1.0
35	28.5	15.3	10.6	3.0	30.0	1.0
36	26.4	14.6	10.4	2.9	36.6	1.0
37	24.4	14.0	10.2	2.9	44.6	1.0
38	22.6	13.4	10.0	2.8	54.4	1.0
39	20.9	12.8	9.8	2.8	66.4	1.0
40	19.3	12.2	9.6	2.8	81.0	1.0
41	18.7	11.8	9.4	2.7	75.5	1.3
42	18.1	11.4	9.2	2.7	70.4	1.6
43	17.5	11.0	9.0	2.6	65.7	2.1
44	17.0	10.6	8.8	2.6	61.3	2.6
45	16.4	10.2	8.6	2.6	57.2	3.4
46	15.9	9.8	8.4	2.5	53.3	4.3
47	15.4	9.5	8.3	2.5	49.7	5.5
48	14.9	9.1	8.1	2.4	46.4	7.0
49	14.4	8.8	7.9	2.4	43.3	8.9
50	14.0	8.5	7.8	2.4	40.4	11.3
51	13.6	8.5	7.6	2.3	39.7	14.4

52	13.1	8.6	7.4	2.3	39.1	18.4
53	12.7	8.7	7.3	2.3	38.4	23.5
54	12.3	8.7	7.1	2.2	37.8	29.9
55	12.0	8.8	7.0	2.2	37.2	28.2
56	11.6	8.8	6.8	2.2	36.6	26.6
57	11.2	8.9	6.7	2.1	36.0	25.2
58	10.9	8.9	6.6	2.1	35.4	23.8
59	10.5	9.0	6.4	2.1	34.8	18.6
60	10.2	9.1	6.3	2.0	34.2	14.5
61	9.9	9.4	6.2	2.0	33.7	11.4
62	9.6	9.8	6.0	2.0	33.1	8.9
63	9.3	10.2	5.9	2.0	32.6	7.0
64	9.0	10.7	5.8	1.9	32.0	5.4
65	8.7	11.1	5.7	1.9	31.5	4.3
66	8.5	11.6	5.6	1.9	31.0	3.3
67	8.2	12.1	5.4	1.8	30.5	2.6
68	7.9	12.6	5.3	1.8	30.0	2.0
69	7.7	13.1	5.2	1.8	29.5	1.6
70	7.5	13.1	5.1	1.8	29.0	1.6
71	7.2	13.1	5.0	1.7	28.5	1.6
72	7.0	13.1	4.9	1.7	28.1	1.6
73	6.8	13.1	4.8	1.7	27.6	1.6
74	6.6	13.1	4.7	1.7	27.2	1.6
75	6.4	13.1	4.6	1.6	26.7	1.6
76	6.2	13.1	4.5	1.6	26.3	1.6
77	6.0	13.1	4.4	1.6	25.9	1.6
78	5.8	13.1	4.3	1.6	25.4	1.6
79	5.6	13.1	4.2	1.5	25.0	1.6

Table S3 Number of ABCFR relatives found to be have PVs by the diagnosis age and PV status of the proband a

Gene	Number of relatives and families		Age groups of the probands (years)											
			Case families						Control families					
			<30	30-39	40-49	50-59	60-69	Total	<30	30-39	40-49	50-59	60-69	Total
BRCA1	Number of relatives		1	27	22	3	0	53	0	1	2	0	0	3
	Number of families where the relatives come from	Probands with PVs	1	17	9	1	0	28	0	1	1	0	0	2
		Probands without PVs	0	0	1	1	0	2	0	0	0	0	0	0
		Probands untested	0	0	0	0	0	0	0	0	0	0	0	0
BRCA2	Number of relatives		4	25	7	5	0	41	0	0	0	0	0	0
	Number of families where the relatives come from	Probands with PVs	3	14	2	0	0	19	0	0	0	0	0	0
		Probands without PVs	0	0	3	2	0	5	0	0	0	0	0	0
		Probands untested	0	2	0	0	0	2	0	0	0	0	0	0
PALB2	Number of relatives		3	2	7	1	0	13	0	0	0	0	0	0
	Number of families where the relatives come from	Probands with PVs	1	1	3	1	0	6	0	0	0	0	0	0
		Probands without PVs	0	0	2	0	0	2	0	0	0	0	0	0
		Probands untested	0	0	0	0	0	0	0	0	0	0	0	0
CHEK2	Number of relatives		4	6	6	2	0	18	0	0	0	0	0	0
	Number of families where the relatives come from	Probands with PVs	2	4	4	0	0	10	0	0	0	0	0	0
		Probands without PVs	0	1	0	2	0	3	0	0	0	0	0	0
		Probands untested	0	0	0	0	0	0	0	0	0	0	0	0
ATM	Number of relatives		0	3	3	7	1	14	0	1	0	1	0	2
	Number of families where the relatives come from	Probands with PVs	0	0	1	2	1	4	0	0	0	1	0	1
		Probands without PVs	0	3	2	2	0	7	0	1	0	0	0	1
		Probands untested	0	0	0	0	0	0	0	0	0	0	0	0

[a] 2 relatives had a PV in two genes: case families – one relative (proband aged 30-39 years) in *BRCA2* and *CHEK2*; control families – one relative (proband aged 30-39 years) in *BRCA1* and *ATM*

Table S4 Number of relatives diagnosed with breast or ovarian cancer by study and the diagnosis age and PV status of the proband

PV status	Relative type	Cancer site	ABCFR case families							ABCFR control families							SEARCH						
			Age group (years)					Total		Age group (years)					Total		Age group (years)						Total
			<30	30-39	40-49	50-59	60-69			<30	30-39	40-49	50-59	60-69			<30	30-39	40-49	50-59	60-69	70-79	
Probands with *BRCA1* PVs	1st-degree	Breast	0	27	7	3	0	37		0	0	2	0	0	2		2	6	14	12	4	0	38
		Ovary	0	4	3	0	0	7		0	0	0	0	0	0		0	3	4	5	4	0	16
	2nd-degree	Breast	4	17	5	0	0	26		0	1	1	0	0	2		1	5	9	3	1	0	19
		Ovary	0	6	1	0	0	7		0	0	1	0	0	1		0	2	5	1	0	0	8
Probands with *BRCA2* PVs	1st-degree	Breast	1	9	1	1	0	12		0	1	0	0	0	1		0	7	25	34	15	0	81
		Ovary	0	0	0	1	0	1		0	1	0	0	0	1		0	1	4	8	3	0	16
	2nd-degree	Breast	6	11	3	1	0	21		0	1	0	0	0	1		1	11	9	7	1	0	29
		Ovary	0	1	0	0	0	1		1	1	0	0	0	2		0	2	1	1	1	0	5
Probands with *PALB2* PVs	1st-degree	Breast	0	3	1	2	0	6		0	0	0	0	0	0		0	0	3	8	6	0	17
		Ovary	0	0	0	0	0	0		0	0	0	0	0	0		0	0	1	1	0	0	2
	2nd-degree	Breast	3	2	1	2	0	8		0	1	0	0	0	1		0	0	3	0	1	0	4
		Ovary	0	0	0	1	0	1		0	0	0	0	0	0		0	0	1	0	0	0	1
Probands with *CHEK2* PVs	1st-degree	Breast	0	1	0	1	0	2		0	0	2	0	0	2		1	0	9	22	8	0	40
		Ovary	0	0	0	0	0	0		0	0	0	0	0	0		0	0	0	2	2	0	4
	2nd-degree	Breast	2	4	0	0	0	6		0	0	0	0	0	0		1	1	5	6	3	0	16
		Ovary	1	0	1	0	0	2		0	0	0	0	0	0		0	0	1	1	1	0	3
Probands with *ATM* PVs	1st-degree	Breast	0	0	0	0	0	0		0	0	0	0	0	0		0	0	7	13	7	0	27
		Ovary	0	0	0	0	0	0		0	0	0	0	0	0		0	0	1	2	3	0	6
	2nd-degree	Breast	0	1	2	0	0	3		0	1	0	0	0	1		0	0	6	0	3	0	9
		Ovary	0	0	0	0	0	0		0	0	0	0	0	0		0	0	0	0	1	0	1
		Breast	0	2	0	1	0	3		0	0	0	0	0	0		1	0	3	0	0	0	4

Probands with *TP53* PVs	1st-degree	Ovary	1	0	0	0	0	1		0	0	0	0	0	0		0	0	0	0	0	0	0
	2nd-degree	Breast	0	9	0	1	0	10		0	0	0	0	0	0		0	0	1	0	0	0	1
		Ovary	0	0	0	0	0	0		0	0	0	0	0	0		0	0	0	0	0	0	0
Probands without PVs	1st-degree	Breast	8	63	39	45	4	159		2	16	17	14	8	57		3	64	353	717	676	1	1814
		Ovary	0	10	6	5	1	22		0	7	6	4	0	17		1	7	44	87	62	0	201
	2nd-degree	Breast	15	184	97	94	12	402		10	92	48	36	9	195		1	65	217	301	169	1	754
		Ovary	1	16	6	8	0	31		3	7	5	2	1	18		0	8	23	28	11	0	70
Probands untested	1st-degree	Breast	1	11	3	0	2	17		0	3	1	0	1	5		1	25	91	137	83	0	337
		Ovary	0	1	0	0	0	1		0	0	0	0	0	0		1	4	12	13	7	0	37
	2nd-degree	Breast	2	21	2	4	0	29		2	7	3	4	5	21		2	20	33	45	22	0	122
		Ovary	1	2	0	0	0	3		0	2	0	0	1	3		0	0	7	1	2	0	10

Table S5 Models including BRCA1, BRCA2, PALB2, CHEK2, ATM, a sixth hypothetical gene and a polygenic component

Parameter	Polygenic component only	*BRCA1, BRCA2* and a polygenic component	*BRCA1, BRCA2, PALB2, CHEK2, ATM* and a polygenic component	With a sixth hypothetical gene: Dominant inheritance model	With a sixth hypothetical gene: Recessive inheritance model	With a sixth hypothetical gene: General inheritance model	With a sixth hypothetical gene: Dominant inheritance model with an age-constant $\sigma_P^2(t)$
$\sigma_P^2(t)$ (95% CI) [a]	α = 5.442 (95% CI: 4.447, 6.438), β = -0.062 (95% CI: -0.079, -0.046)	α = 2.934 (95% CI: 1.854, 4.015), β = -0.024 (95% CI: -0.043, -0.005)	α = 2.814 (95% CI: 1.698, 3.929), β = -0.023 (95% CI: -0.042, -0.003)	α = 2.303 (95% CI: 1.133, 3.472), β = -0.014 (95% CI: -0.035, 0.007)	α = 1.397 (95% CI: 0.257, 2.537), β = -0.002 (95% CI: -0.022, 0.018)	α = 2.291 (95% CI: 1.121, 3.460), β = -0.014 (95% CI: -0.035, 0.007)	1.530 (1.373, 1.696)
PV allele frequency (95% CI)							
BRCA1	N/A	0.082% (0.071%, 0.094%)	0.081% (0.070%, 0.093%)	0.080% (0.069%, 0.092%)	0.080% (0.069%, 0.093%)	0.080% (0.069%, 0.092%)	0.080% (0.069%, 0.092%)
BRCA2	N/A	0.142% (0.126%, 0.159%)	0.141% (0.126%, 0.159%)	0.141% (0.126%, 0.159%)	0.141% (0.126%, 0.158%)	0.141% (0.126%, 0.159%)	0.141% (0.126%, 0.159%)
PALB2	N/A	N/A	0.060% (0.049%, 0.073%)	0.060% (0.049%, 0.073%)	0.060% (0.049%, 0.073%)	0.060% (0.049%, 0.073%)	0.060% (0.049%, 0.073%)
CHEK2	N/A	N/A	0.385% (0.338%, 0.438%)	0.385% (0.338%, 0.439%)	0.385% (0.338%, 0.438%)	0.385% (0.338%, 0.439%)	0.385% (0.338%, 0.438%)
ATM	N/A	N/A	0.167% (0.139%, 0.200%)	0.167% (0.139%, 0.200%)	0.167% (0.139%, 0.200%)	0.167% (0.139%, 0.200%)	0.167% (0.139%, 0.200%)
Hypothetical gene	N/A	N/A	N/A	0.002% (0.001%, 0.006%)	10.6% (0.5%, 21.1%)	0.003% (0.001%, 0.006%)	0.003% (0.001%, 0.008%)
RR of hypothetical gene (95% CI)							
Heterozygote	N/A	N/A	N/A	415.87 (283.55, 609.93)	1	398.63 (168.71, 941.89)	340.15 (143.03, 808.94)
Homozygote	N/A	N/A	N/A	415.87 (283.55, 609.93)	11.61 (4.25, 31.71)	36641.33 (0, 1.3×10^{16})	340.15 (143.03, 808.94)
Log-likelihood	-48094.17	-46507.53	-35414.41	-35406.04	-35410.62	-35406.05	-35406.76
Number of parameters estimated	2	4	7	9	9	10	8
Akaike Information Criterion	96192.34	93031.06	70842.82	70830.08	70839.24	70832.10	70829.52
P [b]	N/A	N/A	N/A	2.3×10^{-4}	0.02	8.1×10^{-4}	9.2×10^{-5}
Best fitting model							Yes

[a] $\sigma_P^2(t) = \alpha + \beta \times$ age

[b] From the likelihood ratio test of comparing with the model including *BRCA1*, *BRCA2*, *PALB2*, *CHEK2*, *ATM* and an age-dependent $\sigma_P^2(t)$

Table S6 Age-specific polygenic variance after fitting major genes and age-specific proportion of breast cancer familial variance explained by major genes

Age group (years)	$\sigma_P^2(t)$ (95% CI) [a]					Proportion of breast cancer familial variance explained by major genes [d]			
	Only a polygenic component fitted [b]	*BRCA1, BRCA2* fitted [b]	*BRCA1, BRCA2, PALB2, CHEK2, ATM* fitted [b]	*BRCA1, BRCA2, PALB2, CHEK2, ATM, TP53* fitted [b]	*BRCA1, BRCA2, PALB2, CHEK2, ATM, TP53*, a seventh hypothetical gene fitted [c]	*BRCA1, BRCA2*	*BRCA1, BRCA2, PALB2, CHEK2, ATM*	*TP53*	The seventh hypothetical gene
20-29	3.869 (3.274, 4.465)	2.340 (1.723, 2.959)	2.245 (1.609, 2.883)	2.108 (1.472, 2.745)	1.272 (0.944, 1.649)	39.52%	41.98%	3.54%	21.59%
30-39	3.240 (2.805, 3.679)	2.102 (1.670, 2.538)	2.017 (1.572, 2.466)	1.920 (1.474, 2.370)	1.272 (0.944, 1.649)	35.12%	37.74%	2.99%	20.00%
40-49	2.611 (2.611, 2.902)	1.864 (1.599, 2.131)	1.790 (1.518, 2.063)	1.733 (1.460, 2.008)	1.272 (0.944, 1.649)	28.60%	31.46%	2.17%	17.65%
50-59	1.982 (1.803, 2.162)	1.626 (1.462, 1.789)	1.562 (1.398, 1.724)	1.546 (1.380, 1.709)	1.272 (0.944, 1.649)	17.95%	21.19%	0.83%	13.79%
60-69	1.353 (1.164, 1.543)	1.389 (1.152, 1.626)	1.334 (1.096, 1.573)	1.358 (1.121, 1.596)	1.272 (0.944, 1.649)	0%	1.37%	0%	6.35%
70-79	0.724 (0.417, 1.032)	1.151 (0.753, 1.550)	1.107 (0.701, 1.514)	1.171 (0.768, 1.575)	1.272 (0.944, 1.649)	0%	0%	0%	0%

[a] For each age group, the variance was assumed to be the variance at the middle point age

[b] From the model in which $\sigma_P^2(t)$ was linearly decreased with age

[c] From the model in which $\sigma_P^2(t)$ was independent with age

[d] For a gene, the age-specific proportion explained by the gene was calculated as the age-specific difference in $\sigma_P^2(t)$ between the model without that gene and the model with that gene divided by the age-specific total breast cancer variance (i.e., column 2). The proportion explained by *BRCA1* and *BRCA2* was calculated as (column 2 – column 3)/column 2, the proportion explained by *BRCA1, BRCA2, PALB2, CHEK2* and *ATM* was calculated as (column 2 – column 4)/column 2, the proportion explained by *TP53* was calculated as (column 4 – column 5)/column 2, the proportion explained by the seventh hypothetical gene was calculated as (column 5 – column 6)/column 2. Where the proportion explained by a gene was negative in older ages, the proportion was assumed to be zero.

Table S7 Families with the largest change in log-likelihood in favour of the best fitting dominant inheritance model of the hypothetical gene after fitting BRCA1, BRCA2, PALB2, CHEK2, ATM and a polygenic component

Family ID	Change in log-likelihood [a]	Age at breast cancer diagnosis (years)					PV in other genes not considered in the best fitting dominant inheritance model of the hypothetical gene
		Proband	Mother	Sisters	Aunts	Grandmothers	
Family 1	5.895	36		30, 36	27 (paternal), 28 (paternal)	35	*TP53*
Family 2	2.983	29	34	34			
Family 3	1.710	39			27 (paternal), 32(paternal)		*TP53*
Family 4	1.477	39	29	24			
Family 5	1.123	24	29				*TP53*
Family 6	0.885	40		20, 23			
Family 7	0.628	27	35				
Family 8	0.497	29	34				
Family 9	0.374	36		33			
Family 10	0.328	41	36		35		

[a] The log-likelihood of the best fitting dominant inheritance model of the hypothetic gene minus the log-likelihood of the model including *BRCA1*, *BRCA2*, *PALB2*, *CHEK2*, *ATM* and an age-constant $\sigma_P^2(t)$

Table S8 Models including BRCA1, BRCA2, PALB2, CHEK2, ATM, TP53 and a polygenic component

**Parameter**	**Age-constant *TP53* RR** [a]	***TP53* RR varied by every 10 years**	***TP53* log-RR as a linear function of age** [b]	***TP53* log-RR as a piecewise function of age** [c]	***TP53* log-RR as a linear function of age in age 20-49 years and constant in age >49 years, with an age-dependent $\sigma_P^2(t)$** [d]	***TP53* log-RR as a linear function of age in age 20-49 years and constant in age >49 years, with an age-constant $\sigma_P^2(t)$** [e]
$\sigma_P^2(t)$ (95% CI) [f]	α = 2.662 (95% CI: 1.593, 3.731), β = -0.020 (95% CI: -0.039, -0.001)	α = 2.585 (95% CI: 1.424, 3.746), β = -0.019 (95% CI: -0.039, 0.002)	α = 2.600 (95% CI: 1.440, 3.761), β = -0.019 (95% CI: -0.040, 0.001)	α = 2.565 (95% CI: 1.445, 3.686), β = -0.019 (95% CI: -0.038, 0.001)	α = 2.576 (95% CI: 1.473, 3.679), β = -0.019 (95% CI: -0.038, 0.001)	1.546 (1.389, 1.711)
PV allele frequency (95% CI)						
BRCA1	0.080% (0.069%, 0.092%)	0.080% (0.069%, 0.092%)	0.080% (0.069%, 0.092%)	0.080% (0.069%, 0.092%)	0.080% (0.069%, 0.092%)	0.080% (0.069%, 0.092%)
BRCA2	0.141% (0.126%, 0.158%)	0.141% (0.126%, 0.158%)	0.141% (0.126%, 0.158%)	0.141% (0.126%, 0.158%)	0.141% (0.126%, 0.158%)	0.141% (0.126%, 0.158%)
PALB2	0.060% (0.049%, 0.073%)	0.060% (0.049%, 0.073%)	0.060% (0.049%, 0.073%)	0.060% (0.049%, 0.073%)	0.060% (0.049%, 0.073%)	0.060% (0.049%, 0.073%)
CHEK2	0.385% (0.338%, 0.439%)	0.385% (0.338%, 0.438%)	0.385% (0.338%, 0.438%)	0.385% (0.338%, 0.438%)	0.385% (0.338%, 0.438%)	0.385% (0.338%, 0.438%)
ATM	0.167% (0.139%, 0.200%)	0.167% (0.139%, 0.200%)	0.167% (0.139%, 0.200%)	0.167% (0.139%, 0.200%)	0.167% (0.139%, 0.200%)	0.167% (0.139%, 0.200%)
TP53	0.007% (0.005%, 0.011%)	0.017% (0.009%, 0.034%)	0.018% (0.008%, 0.041%)	0.017% (0.009%, 0.045%)	0.018% (0.009%, 0.036%)	0.017% (0.009%, 0.034%)
RR of *TP53* PVs (95% CI)						
Age 20-29 years	36.61 (19.96, 67.17)	132.61 (55.40, 317.40)	95.60 (41.19, 222.06)	170.60 (70.65, 412.55)	135.75 (61.60, 298.58)	144.17 (66.24, 311.66)
Age 30-39 years	36.61 (19.96, 67.17)	30.26 (13.34, 68.65)	29.83 (13.01, 67.93)	24.03 (9.74, 59.30)	30.59 (15.06, 62.38)	32.13 (16.08, 64.43)
Age 40-49 years	36.61 (19.96, 67.17)	7.25 (3.07, 17.14)	9.31 (3.89, 25.52)	6.92 (2.40, 19.77)	6.89 (2.48, 19.12)	7.16 (2.66, 19.24)
Age 50-59 years	36.61 (19.96, 67.17)	3.07 (0.28, 33.04)	2.90 (0.79, 10.81)	2.56 (0.65, 10.11)	2.95 (1.09, 7.95)	3.08 (1.16, 8.17)
Age 60-69 years	36.61 (19.96, 67.17)	3.20 (0.85, 12.04)	0.91 (0.17, 4.86)	2.91 (0.69, 12.18)	2.95 (1.09, 7.95)	3.08 (1.16, 8.17)
Age 70-79 years	36.61 (19.96, 67.17)	3.55 (0, 3218679)	0.28 (0.04, 2.24)	4.28 (0.25, 70.11)	2.95 (1.09, 7.95)	3.08 (1.16, 8.17)
Log-likelihood	-35656.36	35634.98	-35638.89	-35635.99	-35635.77	-35637.37

Number of parameters estimated	9	14	10	15	11	10
Akaike Information Criterion	71330.72	71297.96	71297.78	71298.86	71293.54	71294.74
P [g]	N/A	4.1×10^{-8}	3.4×10^{-9}	7.9×10^{-8}	1.1×10^{-9}	7.2×10^{-10}
Best fitting model						Yes

[a] $P<10^{-15}$ from comparing the model with its equivalent nested model including five major genes and an age-dependent polygenic component. Note that, the log-likelihood of the model including five major genes and an age-decreasing polygenic component in Table S4 is not directly comparable with the log-likelihood of the model additionally including *TP53*, because the two models included different numbers of variables. By fixing the *TP53* allele frequency to be zero and the *TP53* RR to be 1 in the model additionally including *TP53*, the model with an age-dependent $\sigma_P^2(t)$ (essentially the same as the model including five major genes and an age-dependent $\sigma_P^2(t)$) had a log-likelihood of -36210.80 and an AIC of 72435.60.

[b] Log-RR = $\alpha + \beta \times (\text{age} - 20)$ in age 20-79 years, where $\alpha = 5.22$ (95% CI: 4.32, 6.12), $\beta = -0.12$ (95% CI: -0.16, -0.08)

[c] Log-RR = $\alpha + \beta_1 \times (\text{age} - 20)$ in age 20-29 years, $\alpha + 10 \times \beta_1 + \beta_2 \times (\text{age} - 30)$ in age 30-39 years, $\alpha + 10 \times (\beta_1 + \beta_2) + \beta_3 \times (\text{age} - 40)$ in age 40-49 years, $\alpha + 10 \times (\beta_1 + \beta_2 + \beta_3) + \beta_4 \times (\text{age} - 50)$ in age 50-59 years, $\alpha + 10 \times (\beta_1 + \beta_2 + \beta_3 + \beta_4) + \beta_5 \times (\text{age} - 60)$ in age 60-69 years, and $\alpha + 10 \times (\beta_1 + \beta_2 + \beta_3 + \beta_4 + \beta_5) + \beta_6 \times (\text{age} - 70)$ in age 70-79 years, where $\alpha = 6.33$ (95% CI: 4.91, 7.75), $\beta_1 = -0.23$ (95% CI: -0.40, -0.05), $\beta_2 = -0.17$ (95% CI: -0.30, -0.04), $\beta_3 = -0.08$ (95% CI: -0.22, 0.06), $\beta_4 = -0.12$ (95% CI: -0.32, 0.08), $\beta_5 = 0.15$ (95% CI: -0.12, 0.41), $\beta_6 = -0.07$ (95% CI: -0.67, 0.53)

[d] Log-RR = $\alpha + \beta \times (\text{age} - 20)$ in age 20-49 years, where $\alpha = 5.66$ (95% CI: 4.69, 6.62), $\beta = -0.15$ (95% CI: -0.21, -0.09)

[e] Log-RR = $\alpha + \beta \times (\text{age} - 20)$ in age 20-49 years, where $\alpha = 5.72$ (95% CI: 4.78, 6.66), $\beta = -0.15$ (95% CI: -0.21, -0.09)

[f] $\sigma_P^2(t) = \alpha + \beta \times \text{age}$

[g] From the likelihood ratio test of comparing with the model including an age-constant *TP53* RR

Table S9 Families with the largest change in log-likelihood in favour of the best fitting recessive inheritance model of the hypothetical gene after fitting BRCA1, BRCA2, PALB2, CHEK2, ATM, TP53 and a polygenic component

Family ID	Change in log likelihood [a]	Age at breast cancer diagnosis (years)				
		Proband	Mother	Sisters	Aunts	Grandmothers
Family 1	0.676	29	34	34		
Family 2	0.519	50	32	39, 45		
Family 3	0.486	31	46	37		
Family 4	0.485	38		42, 46	66 (maternal)	
Family 5	0.484	43		30, 30		
Family 6	0.472	62	54	44, 51, 53		54 (paternal)
Family 7	0.462	47		37, 42		
Family 8	0.437	45	46	38		36 (paternal)
Family 9	0.362	48		45, 50		
Family 10	0.329	46		45		

[a] The log-likelihood of the best fitting recessive inheritance model of the hypothetical gene minus the log-likelihood of the model including *BRCA1*, *BRCA2*, *PALB2*, *CHEK2*, *ATM*, *TP53* and an age-constant $\sigma_P^2(t)$

Table S10 Sensitivity analyses results of assuming the pathogenic variant test sensitivity to be 80% for models including BRCA1, BRCA2, PALB2, CHEK2, ATM, TP53, a polygenic component, with or without a hypothetical gene

Parameters	Without the hypothetical gene	With the hypothetical gene			
	Age-constant $\sigma_P^2(t)$	Dominant inheritance model	Recessive inheritance model [a]	General inheritance model	Recessive inheritance model with an age-dependent $\sigma_P^2(t)$ [b]
$\sigma_P^2(t)$ (95% CI)	1.493 (1.336, 1.658)	1.486 (1.330, 1.652)	1.252 (0.950, 1.595)	1.252 (0.935, 1.615)	α = 0.742 (95% CI: -0.739, 2.223), β = 0.009 (95% CI: -0.016, 0.034)
PV allele frequency (95% CI)					
BRCA1	0.089% (0.078%, 0.103%)	0.089% (0.077%, 0.103%)	0.089% (0.077%, 0.103%)	0.089% (0.077%, 0.103%)	0.089% (0.077%, 0.103%)
BRCA2	0.157% (0.140%, 0.177%)	0.157% (0.140%, 0.177%)	0.157% (0.140%, 0.176%)	0.157% (0.140%, 0.176%)	0.157% (0.140%, 0.176%)
PALB2	0.067% (0.055%, 0.082%)	0.067% (0.055%, 0.082%)	0.067% (0.055%, 0.081%)	0.067% (0.055%, 0.081%)	0.067% (0.055%, 0.081%)
CHEK2	0.432% (0.379%, 0.492%)	0.432% (0.379%, 0.492%)	0.432% (0.379%, 0.492%)	0.432% (0.379%, 0.492%)	0.432% (0.379%, 0.492%)
ATM	0.187% (0.156%, 0.224%)	0.187% (0.156%, 0.224%)	0.187% (0.156%, 0.224%)	0.187% (0.156%, 0.224%)	0.187% (0.156%, 0.224%)
TP53	0.020% (0.010%, 0.039%)	0.020% (0.008%, 0.051%)	0.020% (0.010%, 0.041%)	0.020% (0.010%, 0.042%)	0.020% (0.009%, 0.043%)
Hypothetical gene	N/A	0.001% (0.0001%, 0.006%)	12.6% (5.4%, 21.8%)	12.6% (5.2%, 22.5%)	12.7% (6.2%, 19.6%)
RR of *TP53* PVs (95% CI)					
Age 20-29 years	138.06 (64.62, 294.21)	132.33 (54.36, 320.67)	138.66 (60.99, 313.92)	138.66 (59.76, 320.30)	144.85 (59.03, 353.78)
Age 30-39 years	31.13 (15.55, 62.59)	30.30 (12.32, 74.73)	31.32 (15.02, 65.66)	31.32 (14.55, 67.78)	32.41 (14.39, 73.28)
Age 40-49 years	7.02 (2.58, 19.12)	6.94 (2.05, 23.47)	7.07 (2.57, 19.43)	7.07 (2.50, 20.02)	7.25 (2.52, 20.95)
Age 50-59 years	2.99 (1.08, 8.30)	2.93 (0.56, 15.37)	3.03 (0.98, 9.37)	3.03 (0.89, 10.33)	3.12 (0.87, 11.21)
Age 60-69 years	2.99 (1.08, 8.30)	2.93 (0.56, 15.37)	3.03 (0.98, 9.37)	3.03 (0.89, 10.33)	3.12 (0.87, 11.21)
Age 70-79 years	2.99 (1.08, 8.30)	2.93 (0.56, 15.37)	3.03 (0.98, 9.37)	3.03 (0.89, 10.33)	3.12 (0.87, 11.21)

RR of the hypothetical gene (95% CI)					
Heterozygote	N/A	367.24 (308.71, 436.87)	1	1	1
Homozygote	N/A	367.24 (308.71, 436.87)	9.69 (4.09, 22.98)	9.69 (4.06, 23.12)	10.59 (5.01, 22.38)
Log-likelihood	-35636.08	-35635.20	-35631.95	-35631.95	-35631.74
Number of parameters estimated	10	12	12	13	13
Akaike Information Criterion	71292.16	71294.4	71287.90	71289.90	71289.48
P [c]	N/A	0.41	0.02	0.04	0.03
Best fitting model			Yes		

[a] Log-RR = $\alpha + \beta \times (\text{age} - 20)$ in age 20-49 years, where $\alpha = 5.68$ (95% CI: 4.69, 6.66), $\beta = -0.15$ (95% CI: -0.20, -0.09)

[b] $\sigma_P^2(t) = \alpha + \beta \times \text{age}$

[c] From the likelihood ratio test of comparing with the model including *BRCA1*, *BRCA2*, *PALB2*, *CHEK2*, *ATM*, *TP53* and an age-constant $\sigma_P^2(t)$

Supplemental Acknowledgements

The ABCFR was supported in Australia by the National Health and Medical Research Council (NHMRC), the New South Wales Cancer Council, the Victorian Health Promotion Foundation, the Victorian Breast Cancer Research Consortium, Cancer Australia, and the National Breast Cancer Foundation. The six sites of the Breast Cancer Family Registry (BCFR) were supported by grant UM1 CA164920 from the U.S. National Cancer Institute. The content of this manuscript does not necessarily reflect the views or policies of the National Cancer Institute or any of the collaborating centres in the BCFR, nor does mention of trade names, commercial products, or organizations imply endorsement by the U.S. Government or the BCFR. Sequencing of the ABCFR samples was supported by an NHMRC Program grant (APP1074383), The National Breast Cancer Foundation (BRA-STRAP; NT-15-016), NHMRC European Union Collaborative Research Grant (APP1101400) and Monash University, Melbourne, Australia. SEARCH was funded by Cancer Research UK (C490/A16561) and the NIHR Biomedical Research Centre at the University of Cambridge and the University of Cambridge has received salary support for P.D.P.P. from the NHS in the East of England through the Clinical Academic Reserve. Sequencing of the SEARCH samples was funded by European Union's Horizon 2020 Research and Innovation Programme (BRIDGES: grant number 634935).
